# A comparison of topologically associating domain callers over mammals at high resolution

**DOI:** 10.1186/s12859-022-04674-2

**Published:** 2022-04-12

**Authors:** Emre Sefer

**Affiliations:** Department of Computer Science, Ozyegin University, Istanbul, Turkey

**Keywords:** Chromatin organization, Hi-C, Micro-C, Topologically associating domains, TAD callers

## Abstract

**Background:**

Topologically associating domains (TADs) are locally highly-interacting genome regions, which also play a critical role in regulating gene expression in the cell. TADs have been first identified while investigating the 3D genome structure over High-throughput Chromosome Conformation Capture (Hi-C) interaction dataset. Substantial degree of efforts have been devoted to develop techniques for inferring TADs from Hi-C interaction dataset. Many TAD-calling methods have been developed which differ in their criteria and assumptions in TAD inference. Correspondingly, TADs inferred via these callers vary in terms of both similarities and biological features they are enriched in.

**Result:**

We have carried out a systematic comparison of 27 TAD-calling methods over mammals. We use Micro-C, a recent high-resolution variant of Hi-C, to compare TADs at a very high resolution, and classify the methods into 3 categories: feature-based methods, Clustering methods, Graph-partitioning methods. We have evaluated TAD boundaries, gaps between adjacent TADs, and quality of TADs across various criteria. We also found particularly CTCF and Cohesin proteins to be effective in formation of TADs with corner dots. We have also assessed the callers performance on simulated datasets since a gold standard for TADs is missing. TAD sizes and numbers change remarkably between TAD callers and dataset resolutions, indicating that TADs are hierarchically-organized domains, instead of disjoint regions. A core subset of feature-based TAD callers regularly perform the best while inferring reproducible domains, which are also enriched for TAD related biological properties.

**Conclusion:**

We have analyzed the fundamental principles of TAD-calling methods, and identified the existing situation in TAD inference across high resolution Micro-C interaction datasets over mammals. We come up with a systematic, comprehensive, and concise framework to evaluate the TAD-calling methods performance across Micro-C datasets. Our research will be useful in selecting appropriate methods for TAD inference and evaluation based on available data, experimental design, and biological question of interest. We also introduce our analysis as a benchmarking tool with publicly available source code.

## Introduction

Chromosomes store epigenetic and genetic code in the cell, they have complex 3D structure, and they are interweaved into a tiny spatial space in 3D. A chromosome’s 3D conformation is important in genomic organization [1–3] as well as regulation, transcription, and DNA replication [4–8] as shown by a number of up-to-date studies. High-throughput chromatin conformation capture (Hi-C) [9] and Micro-C [10] technologies have been developed to quantitatively measure the number of interactions among all genomic loci pairs throughout the genome via high-throughput sequencing. These high-throughput techniques are derived from chromatin proximity ligation method [11–14]. Among these techniques, Micro-C uses micrococcal nuclease rather than restriction enzymes to partition the chromatin into fragments which enables chromosome folding maps at nucleosome resolution. Micro-C can also be seen as a variant of Hi-C method. More Importantly, we can analyze the chromatin structure in more detail via Micro-C by overcoming the Hi-C experiment’s resolution gap at micro scales. By using Hi-C and Micro-C, multiple species’ genome structures have been investigated. For instance, yeast [10, 15, 16], drosophila [2, 17], plants [18, 19], and mammals [1, 3, 9, 20, 21].

Chromosomal regions identified by these datasets favorably interact with each other, resulting in high-order structural regions such as topologically associating domains (TADs), compartments, and chromosomal territories. These constituent structures have different sizes, and are related with different molecular features in the genome [1, 2, 22, 23]. For instance, TADs are chromosomal regions with almost megabase size which self-interact more frequently than the other chromosomal regions [1–3]. One of the most important characteristic of TADs is their demarcation via boundaries. TADs are in general found to be highly conserved, relatively stable across cell types and species [1, 24], even though such notion that TADs are highly conserved across cell types has been questioned recently [25, 26] including single cell studies showing extensive variability in TAD-like structures [27–30]. TADs possess important biological functions such as them having relationship with cancers and genetic disorders [31–35]. TADs are also stable during replication-timing regulation [36] and cell differentiation. Even though the link between gene expression changes and TADs has recently been debated [37–45], TADs are associated with developmental processes [46]. Therefore, accurate identification of TADs is important in linking the cellular function to the spatial genome organization.

Recently, subTADs have been discovered which are sub-megabased size, smaller domains in the chromatin. These subTADs are nested hierarchically inside TADs across mammalian Hi-C interaction maps [3, 47, 48]. The large fraction of nested subTADs are missing in the low resolution Hi-C data. However, almost all of them can be easily identified at genome-wide scale after creation of better techniques that allow us to generate genome-wide architecture maps at a high resolution (1–4 kb). The demarcation by boundaries is also observed in subTADs so their domain structure looks like TADs. Even though the existence of similar demarcation, insulation strength is weaker in subTAD boundaries since they can attenuate long distance interactions among domains at a lower capacity. SubTADs also show cell-type-dynamic folding characteristics more than TADs [1, 47].

CTCF binding sites are enriched at TAD boundaries [49–55] in addition to cohesin proteins SCM3 and RAD21 [55]. Many other functional elements are also enriched at TAD boundaries. For instance, RNA polymerase II, short interspersed nuclear elements (SINEs), housekeeping genes, promoter-associated histone marks, and transcription start sites (TSSs) [1, 2, 56, 57]. Particularly, H3K9me3 is a histone mark associated with non-promoters and TAD boundaries are depleted in terms of H3K9me3 [1]. These enrichments and depletions show the importance of TADs, as well as their importance in the genome at various angles. In addition, TADs sometimes consist of subTADs indicating their hierarchical structure [58–60].

TAD inference has become an important promising research topic in the last decace since their discovery by Dixon et al. [1]. Dixon et al. [1] has come up with the TAD concept and has developed DI (directionality index) technique to detect TADs. Afterwards, many computational TAD identification techniques (TAD callers) have been developed. The development of Hi-C techniques is important from genome research perspective since it has opened a new era while promoting many characteristics of TADs. In this case, relative analysis and comparison of the developed TAD callers is quite important in promoting faster enhancement in TAD identification. Such comparison will be helpful to researchers in customizing their methods according to available design and datasets. Only in last two years, many techniques have been proposed to detect TADs across different species. These novel methods include the most recent TADBD, SpectralTAD, OnTAD, Constrained HAC, MSTD, GRiNCH, and deDoc [1, 3, 56, 57, 59–85]. Up-to-now, there are almost 31 TAD callers and Table 1 summarizes the characteristics of these methods.

**Table 1:** The comparison of the evaluated TAD caller features

TAD Caller	Runtime complexity	Hierarchical TADs	Gaps Allowed	Parameters	Input Details	Implementation Language
3DNetMod [76]	*O*(*n*)	✓	✓	18	Sparse 3-column matrix	Python
Armatus [56]	$O(tn^2)$	✓	✓	1	$n \times n$ matrix	C++
Arrowhead [3]	$O(n^2)$	✓	✓	1	.hic file	Java
CaTCH [60]	NA	✓	✓	1	Sparse 4-column matrix	R
Constrained HAC [78]	$O(n(h+\log (n)))$	✗	✗	1	$n \times n$ matrix	R
CHDF [64]	NA	✗	✗	1	$n \times n$ matrix	C++
chromoR [62]	NA	✗	✗	2	$n \times n$ matrix	R
ClusterTAD [67]	NA	✗	✓	1	$n \times n$ matrix	MATLAB
deDoc [74]	$O(n\log ^{2}n)$	✓	✓	0	Sparse 3-column matrix	Java
DI [1]	NA	✗	✓	3	$n \times (n + 3)$ matrix	MATLAB, Perl
EAST [68]	$O(n^2)$	✗	✓	3	$n \times n$ matrix	Python
GMAP [73]	NA	✓	✓	10	$n \times n$ matrix	R, C++
GRiNCH [85]	$O(kn^2)$	✗	✓	3	$n \times n$ matrix	C++
HiCExplorer [77]	NA	✗	✓	4	h5 file	Python
HiCKey [84]	$O(n^3)$	✓	✓	3	$n \times n$ matrix	C++
HiCseg [61]	$O(Kn^{2})$	✗	✗	3	$n \times n$ matrix	R, C
HiTAD [71]	NA	✗	✓	1	.cool file	Python
IC-Finder [66]	NA	✗	✓	2	$n \times n$ matrix	MATLAB
InsulationScore [63]	NA	✗	✗	5	$(n + 1) \times (n + 1)$ matrix	Perl
Matryoshka [75]	$O(tl^{2})$	✓	✓	1	$n \times (n + 3)$ matrix	C++
MrTADFinder [72]	$O(n^3)$	✗	✓	1	Sparse 3-column matrix	Julia
MSTD [80]	NA	✗	✓	1	$n \times n$ matrix	Python
OnTAD [79]	$O(md^{2})$	✓	✓	5	$n \times n$ matrix	C++
PSYCHIC [69]	NA	✓	✗	1	$n \times n$ matrix	MATLAB, Python,Perl
Spectral [65]	NA	✗	✓	2	$n \times n$ matrix	MATLAB
SpectralTAD [81]	*O*(*n*)	✓	✓	11	$n \times n$ matrix	R
TADBD [82]	NA	✗	✓	2	$n \times n$ matrix	R
TADbit [70]	NA	✗	✗	1	$n \times n$ matrix	Python, C
TADpole [83]	NA	✓	✓	3	$n \times n$ matrix	R
TADtree [59]	$O(nS^{5})$	✓	✓	6	$n \times n$ matrix	Python
TopDom [57]	NA	✗	✓	1	$n \times (n + 3)$ matrix	R

The parameters below are as follows: Hi-C/Micro-C interaction matrix size is referred by *n*. TADtree’s *S* parameter refers to the maximum size of inferred TADs. OnTAD’s *d* parameter refers to the maximum size of inferred TADs, whereas *m* refers to the expected count of possible boundaries. Matryoshka’s *t* parameter defines the number of resolutions to be inferred, and *l* refers to interval frequency while clustering the inferred *t* resolutions. HiCseg’s *K* parameter defines the maximum number of diagonal TAD partitions. *h* parameter in Constrained HAC is the bandwidth. *k* in GRiNCH is the rank of low-dimensional matrices. Lastly, Armatus’ *t* parameter defines the number of resolutions to be inferred

In this research, we systematically evaluate and compare the performance of 27 TAD identification methods on synthetic data as well as on experimental high-resolution Micro-C interaction datasets. By such comparison, we evaluate these methods as well as their biological and statistical features. We apply the following 3 strategies in our comparison: between the labeled TADs and the inferred TADs on synthetic data, between replicates over the same TAD caller, among callers on the same experimental data. We evaluate the performance of callers by 8 metrics such as Jaccard Index (JI), *p* value ($\le 0.05$) ratio, fold change, boundary tagged ratio, average peak, TAD$adjR^2$, false positive rate (FPR), and true positive rate (TPR). Our analysis consists of the following 3 criteria: TADs and TAD boundaries reproducibility in replicates, depletion and enrichment of functional elements near TAD boundaries, and interaction frequencies decay by the distance in the genome. Moreover, we also focus on the quality of the TAD callers implementation. We have the following main contributions in this research: Expose up-to-date research status in TAD identification which will include the features of different TAD-calling methods as well as potential limits in TAD inference.

This research will complement the previous benchmarking studies [53, 55, 86–88] on Hi-C analysis. It will enhance the reproducibility and robustness of these studies results, and will provide a list of guidelines in designing molecular and computational Hi-C studies. Different than the previous benchmarking studies, we make the following additional contributions: 1- We classify TAD callers into 3 groups: Feature-based methods, Clustering methods, Graph-partitioning methods. We found that feature-based TAD callers in general consistently outperform graph-based and clustering-based TAD callers, 2- We evaluate TAD callers on the recent significantly high-resolution Micro-C datasets instead of lower resolution Hi-C datasets, 3- We evaluate TAD callers across mammals instead of just human, 4- We evaluate the TAD callers performance in terms of inferring corner-dot domains (with extrusion loop) and TADs without corner dots, 5- We also assess the TAD callers performance on the systematically-generated synthetic data at a high resolution where supervised learning is quite difficult, 6- Lastly, we compare TAD callers in detail in terms of eight metrics. In summary, our work provides users with clear guideline to make the best appropriate choice of which TAD callers to use according to their experimental design and available data.

## Materials and methods

### TAD callers

The massive amount of Hi-C experiments that provide interaction counts between pairs of genomic loci have resulted in continuous research on chromatin interactions. As a result of these experiments, we obtain *n*-by-*n* symmetric interaction matrix where each matrix entry represents the number of chromatin interactions between pair of loci. Among the identified genomic structures through these experiments, topologically associating domains (TADs) are one of the most common ones. Practically, TAD callers infer TADs via extracting square diagonal consecutive regions with higher number of interactions. Hierarhical structure of TADs has been observed more recently. As a result, earlier TAD-calling methods cannot detect hierarchical TADs whereas the most recent callers can also detect hierarchical TADs. Even though there is now a plenty of research helping us to understand the nature of TADs, the precise definition of TADs is still missing in terms of hierarchy, size, and number which is one of the major difficulties in TAD analysis. As a result, benchmarks are missing, and TAD accuracy is evaluated indirectly via relating TADs with biological attributes in many cases.

Even though TAD-calling methods simply depend on its contiguous highly self-interacting chromosomal segments definition of TADs, we partition the current TAD callers that combine various dataset features and different techniques into 3 categories as discussed in [88]: 1- Feature-based techniques, 2- Clustering techniques, and 3- Graph-partitioning techniques. Among these techniques, the feature-based techniques in general utilize the patterns and attributes identified in Hi-C data by using common techniques. For instance, they solve for an optimization problem by dynamic programming, and filter out false positives by statistical tests. The clustering techniques in general use more traditional classification and clustering methods, where they focus on clustering contiguous genomic segments into a single cluster by using the similarity values or similarly transitioning states. Lastly, the graph-partioning techniques mostly consider Hi-C interaction frequency matrix as an undirected graph. Then, they partition the graph into subgraphs that correspond to TADs by using graph-based techniques.

Below, we provide a summary of each evaluated TAD-calling method. We have not included 3 TAD callers in our analysis [62, 71, 76] since they have failed to identify TADs in our experiments.

#### Feature-based methods

Filippova et al. [56] introduced an optimization method called Armatus to detect nonoverlapping TADs across multiple scales based on the scaled density, inspired by the observation that TADs may exist at multiple length scales. Armatus detects TADs with a quality function combining the definition of TADs and resolution parameters, and this function is a scaled density of contact frequencies between genomic locus pairs. Armatus uses an $n \times n$ Hi-C matrix (*n* refers to the dimension of the symmetric Hi-C matrix, same below in the present section) as input data and has thirteen parameters. Except for the necessary and optional parameters, the only key parameter in Armatus represents the maximum detection resolution. The time complexity of Armatus is $O(tn^2)$, where *t* refers to the number of resolution parameters that needs to be computed. Armatus outputs a set of overlapped TADs at multiple scales, but these TADs are not coordinated into an ultimate set of hierarchical TADs.

Rao et al. [3] proposed a method called Arrowhead, which applies an ad hoc transformation on the Hi-C contact matrix to highlight TAD borders. After transformation, TADs are visualized as arrowhead shapes whose corner pixels are closely related to three criteria. Then, TADs are extracted on each pixel by dynamic programming. Arrowhead uses .hic files as input data, which can be converted from text files by Juicer software [89] and has one key parameter deciding the sliding window size. The time complexity of Arrowhead is $O(n^2)$. Arrowhead outputs TADs that are qualified by fixed thresholds, which may lead to missing TADs.

Zhan et al. [60] introduced the CaTCH method based on the reciprocal insulation (RI) measure, which estimates the quality of isolation between a TAD and its adjacent TADs. Given a reciprocal insulation threshold, two consecutive TADs whose RIs are smaller than the threshold are merged into a one TAD. CaTCH systematically identifies a set of hierarchical TADs by adjusting the threshold. CaTCH uses a sparse 4-column Hi-C matrix as input data, and its threshold is the key parameter.

Shavit et al. [62] proposed the chromoR method, based on a wavelet change point analysis. chromoR assumes that the Hi-C matrix obeys Poisson distribution. chromoR method calculates 1D contact profile which corresponds to the row sums of the contact matrix, followed by a wavelet Poisson change point detection algorithm. The final detected change points are treated as TAD boundaries. The chromoR method uses an $n \times n$ Hi-C matrix as input data and has two key parameters that are minimal and maximal levels at which the change points are detected.

Roayaei Ardakany et al. [68] proposed the EAST method based on fast 2D convolution of Haarlike features with a scoring function. The summed area table (SAT) calculating the sum of values in a rectangular region is used to assess how well a such region satisfies the features of a TAD. EAST outputs a set of contiguous non-overlapping TADs by optimizing the objective function. EAST accepts an $n \times n$ Hi-C matrix as input data and its time complexity is $O(n^2)$.

Yu et al. [73] introduced the Gaussian Mixture model And Proportion test (GMAP) algorithm to detect TADs. GMAP has three main steps: (1) distinguishing chromatin contacts within and outside a TAD by fitting a two-component Gaussian mixture model, (2) detecting significant boundaries by a proportion test, and (3) identifying TADs based on the boundaries in former step. GMAP identifies a set of hierarchical TADs by recurrently detecting subTADs from TADs. GMAP uses an $n \times n$ Hi-C matrix as input data and has ten key parameters.

Ramírez et al. [77] developed a TAD detection method which is integrated in the HiCExplorer software. HiCExplorer first transforms the Hi-C contact matrix into a *z*-score matrix where each entry is calculated based on the mean and standard deviation of all entries at the same genomic distance. For each bin, the TAD-separation score is computed as the mean of *z*-scores between upstream and downstream regions with a window. TAD-separation scores calculated in multiple window sizes are averaged as the final score for each bin. Like TopDom, a Wilcoxon rank-sum test is used to compare the *z*-scores in each of upstream and downstream regions for the bin, and the highest value of two *p* values is used. By correcting the *p* values using the Bonferroni method, bins with *p* value lower than the threshold is retained. HiCExplorer outputs a set of non-overlapping TADs.

Xing et al. [84] developed a statistical model of TADs called HiCKey, which identifies TADs in two main steps: (1) initially, the method proposes a generalized likelihood-ratio test to detect transition points in given interaction matrix which can follow general mixture or negative binomial distribution, (2) the method runs a number of search strategies to infer hiearchical TADs with their associated *p* values calculated by the generalized likelihood-ratio test. HiCKey can use an $n \times n$ Hi-C matrix as input data and has three key parameters: lower bound of TAD size, threshold to test whether a boundary (change-point) is significant, threshold for identifying nested TADs. The worst-case time complexity of HiCKey is $O(n^3)$.

Lévy-Leduc et al. [61] developed a statistical model of TADs called HiCseg, which identifies TADs with two ending points of diagonal blocks after reducing the 2D segmentation problem to the 1D segmentation problem by maximum likelihood estimation. HiCseg uses an $n \times n$ Hi-C matrix as input data and has three key parameters: the maximal number of change points, the distribution of data, and the type of model. The time complexity of HiCseg is $O(Kn^2)$, where *K* is the maximal number of diagonal blocks. HiCseg assumes that the Hi-C data follow a certain distribution, which may be impractical and sometimes not applicable.

Crane et al. [63] designed a method of turning points, called InsulationScore, which has three main steps: (1) Calculating an insulation score representing the average contact frequencies between upstream and downstream regions with a window, (2) detecting the local minima of the insulation score curve, and (3) filtering out the local minima whose boundary strength is less than 0.1. The local minima are treated as TAD boundaries. InsulationScore uses an $(n+1) \times (n+1)$ Hi-C matrix and five key parameters. InsulationScore relies on the sharply falling contact frequencies around TAD boundaries. However, its fixed parameters are inelastic to filter the false positives.

Malik et al. [75] developed a method named Matryoshka from the Armatus method. Matryoshka obtains a hierarchy of non-overlapping TADs at multiple resolutions. The resolution values are clustered based on the variation of information distance between the corresponding TAD sets and are used to determine the discrete levels of the hierarchy. Matryoshka identifies a set of consensus TADs at each level of hierarchy. Matryoshka uses an $n \times n$ Hi-C matrix as input data. The time complexity of Matryoshka is $O(tl^2)$, where *t* is the number of resolution parameters and *l* is the number of intervals in the step of clustering *t* TAD sets.

An et al. [79] proposed a method of turning points called OnTAD. Another similar method TopDom detects TADs with a fixed window, which is not flexible enough to detect TADs of different sizes and hierarchies. On the other hand, OnTAD captures the candidate boundaries by an adaptive local minimum search approach at multiple window sizes and prunes false positives according to a threshold flexible to the data. Then, TADs are assembled recursively from the candidate boundaries using dynamic programming. OnTAD uses an $n \times n$ Hi-C matrix as input data and has five key parameters. The time complexity of OnTAD is $O(md^2)$, where *m* is the number of candidate boundaries and *d* is the maximum TAD size. The multiple window sizes and flexible filtering of OnTAD enable it to outperform InsulationScore and TopDom, though all of them are based on turning points.

Ron et al. [69] designed a method to identify promoter-enhancer interactions called PSYCHIC. PSYCHIC performs a two-component probabilistic model to obtain the probability of intra- and inter-TAD interactions. Afterwards, it uses a Dynamic Programming algorithm to find the optimal segmentation of each chromosome with a probabilistic score computing the log-posterior ratio of the two sub-models. PSYCHIC uses an $n \times n$ Hi-C matrix as input data and outputs a hierarchical set of TADs. PSYCHIC requires window size as its key parameter.

Lyu et al. [82] introduced a method called TADBD based on Haar feature. TADBD has three main steps: (1) Calculating Haar feature of each point on the diagonal of contact matrix, (2) detecting candidate TAD boundaries by considering multi-scale aggregation at Haar template size, and (3) retaining significant TAD boundaries in the Wilcoxon rank-sum test between the intra-TAD vector and the inter-TAD vector. TADBD uses an $n \times n$ Hi-C matrix as input data and outputs non-overlapping TADs.

Serra et al. [70] developed a breakpoint detection method, called TADbit. TADbit assumes that the contact frequencies follow the Poisson distribution and a power-law decay. TADbit partitions a chromosome into TADs with the optimal log-likelihood which uses the Poisson regression and penalized Bayesian Information Criterion. TADbit accepts an $n \times n$ Hi-C matrix as input data and has one key parameter deciding the maximal TAD size.

Weinreb et al. [59] designed an optimization method that is based on empirical distributions of contact frequencies within TADs, called TADtree. TADtree models hierarchies of nested TADs, including TAD trees and TAD forests, and detects TADs with quality functions calculating the density of contact frequencies between genomic locus pairs based on two ideas: the enrichment of contact frequencies increases at a faster rate within TADs, and the ending points of TADs should have a high boundary index. TADtree uses an $n \times n$ Hi-C matrix as input data and has six key parameters. The time complexity of TADtree is $O(nS^5)$, where *S* is the maximum TAD size. TADtree outputs hierarchical TADs from its complicated model, but its high computational complexity limits its practical application.

Shin et al. [57] developed a method called TopDom based on turning points. TopDom has three steps: (1) calculating binSignal which represents the average contact frequencies between upstream and downstream regions with a window, (2) detecting the local minima of the binSignal curve, and (3) filtering the false-positive minima by testing the difference between interactions and within interactions by the Wilcoxon rank-sum test. Regions between two consecutive local minima are annotated as TADs. Using an $n \times (n+3)$ Hi-C matrix as input data, TopDom has the window size as its single key parameter, and may result in missing TADs of different sizes.

#### Clustering methods

Wang et al. [64] assumed that three kinds of regions exist in a Hi-C contact matrix-domain regions, the regions between two adjacent regions, and the residuals-and developed a clustering method called CHDF. CHDF clusters the contact matrix by dynamic programming with a criterion function combining the sum-of-squared-error and a penalty term related to chromosome contact density. Then, TADs are detected as the two ending points of clusters of domain regions. CHDF uses an $n \times n$ Hi-C matrix as input data and has one key parameter, which is the maximum TAD size.

Oluwadare et al. [67] designed a new method by combining unsupervised learning, called ClusterTAD. TADs are identified as clusters by (1) Roughly estimating the cluster count, (2) Clustering via the K-means approach, and (3) Adjusting through the quality assessment of clusters with chromosome contact frequencies. ClusterTAD uses an $n \times n$ Hi-C matrix as input data and has the maximum TAD size as its only key parameter. ClusterTAD reveals the close similarity of adjacent bins belonging to the same TAD by applying an unsupervised learning approach to TAD calling.

Ambroise et al. [78] developed a fast clustering method based on classical hierarchical agglomerative clustering (HAC), so-called Constrained HAC. Constrained HAC iteratively merges two most similar adjacent clusters into one cluster using HAC with Ward’s linkage [90]. TADs are finally recognized by clustering on the dendrogram with a broken stick model or slope heuristic. Constrained HAC reduces the time and space complexity under a band similarity assumption, which makes it an efficient algorithm on high dimensional data. Constrained HAC accepts an $n \times n$ Hi-C matrix as input data and bandwidth is the key parameter. The time complexity of Constrained HAC is $O(n(h+\log (n)))$, where *h* is the bandwidth.

Dixon et al. [1] developed a clustering method based on the hidden Markov model (HMM), called DI (directionality index). DI calculates the difference of contact frequencies between downstream bins and upstream bins for each bin with a window, which is then applied in HMM to predict each bin to be one of three preset states. TADs are clustered as continuous regions from the beginning bin of the downstream-biased state to the last bin of the upstream-biased state. DI needs an $n \times (n+3)$ Hi-C matrix as input data and has three key parameters. DI was the first method to call TADs and is also used as the benchmarking method to compare the later methods.

Lee et al. [85] developed a clustering method based on constrained non-negative matrix-factorization (NMF), called GRiNCH. GRiNCH is based on NMF, which is a strong dimensionality reduction technique to infer meaningful low-dimensional structures from higher-dimensional data. Strong distance dependence exists in Hi-C data, so they employ a graph-regularized NMF where underlying graph handles the interaction frequencies distance dependence so the inferred low-dimensional structure changes smoothly. GRiNCH can also achieve smoothing out of a sparse interaction matrix by imputing missing matrix entries via NMF. GRiNCH uses an $n \times n$ Hi-C matrix as input data and has three key parameters: *k* as the low-dimensional matrices rank, $\lambda$ which controls the regularization degree, and *r* for neighborhood radius in regularization graph. The worst-case time complexity of GRiNCH is $O(kn^2)$.

Wang et al. [71] presented HiTAD, which is developed from DI. HiTAD computes an adaptive directionality index (DI)-based Hidden Markov Model to generate a genome-wide pool of bottom domains. Regarding the bottom domains as basic elements, TADs are detected by combining the global intra-chromosomal interactions under an objective function. TADs are repeatedly used to call sub-TADs to form a hierarchical set of TADs. HiTAD accepts a cool file as input file and has a key parameter, namely the maximal TAD size.

Haddad et al. [66] developed a method based on the hierarchical clustering approach, called IC-Finder. IC-Finder has three steps: 1- Defining each bin as an individual cluster, 2- Merging the closest clusters of weak heterogeneity into a larger cluster while imposing linear connectivity, and 3- Stopping clustering when clusters of strong heterogeneity remain. IC-Finder uses an $n \times n$ Hi-C matrix as input data and has two key parameters, which are the low and high thresholds to control the merging of two clusters. IC-Finder uses flexible criteria to determine TADs, which is different from the rough estimation of the number of TADs in ClusterTAD.

Ye et al. [80] proposed a fast density-based clustering method called MSTD. MSTD aims to call TADs by clustering points with rectangular shapes, and it has two main steps: 1- Determining cluster centers via the density of chromosome contact frequencies, and 2- Assigning the remaining elements to the same cluster as its nearest-neighbor element of higher density layer by layer. Additionally, MSTD detects promoter-anchored interacting domains (PADs) by working on the promoter-capture Hi-C maps that are asymmetric. MSTD uses an $n \times n$ Hi-C matrix as input data for TAD calling and has one key parameter. MSTD first determines the center of TADs with high local density, which is novel and flexible for adjusting the neighboring bins, but fixed thresholds are still its main limitations.

Soler-Vila et al. [83] developed a clustering method based on combining principal component analysis and constrained hierarchical clustering, called TADpole. TADpole has three main steps: 1- Preprocessing of the input interaction matrix by principal component analysis, 2- Constrained hierarchical clustering optimization, and 3- Genome segmentation. Once distance matrix is formed by the principal components identified in the first step, TADpole than partitions this distance matrix into TADs by utilizing a constrained hierarchical clustering process. TADpole uses an $n \times n$ Hi-C matrix as input data and has three key parameters: the maximum number of principal components to extract, the minimum number of TADs to infer, and the fraction of the interaction matrix which will be tagged as useless columns.

#### Graph partitioning methods

Norton et al. [76] presented the 3DNetMod method using the network modularity after formulating the TAD detection problem as a community detection problem. 3DNetMod applies a Louvain-like, locally greedy algorithm to maximize a given network modularity, and the adjacent nodes belonging to a community are detected as a TAD. 3DNetMod uses a sparse-3 column matrix as input and outputs a hierarchical set of TADs. The time complexity of 3DNetMod is *O*(*n*).

Li et al. [74] formulated the TAD identification problem as a graph-partitioning problem and designed a method called deDoc. deDoc initializes the graph as a coding tree and then performs greedy merging and greedy combining operations with minimal graph structural entropy to minimize the global uncertainty of the Hi-C graph until no operation can be performed. TADs are extracted as the continuous leaf nodes in the final coding tree. deDoc uses a sparse matrix as input data and has no extra key parameter in addition to the necessary parameters. The time complexity of deDoc is $O(n\log ^2n)$. deDoc is the first TAD caller without a parameter, which automatically and naturally partitions TADs from Hi-C data. deDoc outputs hierarchical TADs with two levels.

Yan et al. [72] introduced the MrTADFinder method by formulating the TAD identification as a community detection problem in graph theory. MrTADFinder defines the modularity and objective function in a randomized null model. By optimizing the objective function via the modified Louvain algorithm, MrTADFinder continuously partitions the network into non-overlapping TADs along the chromosome. MrTADFinder accepts sparse 3-column Hi-C matrix as input data, and its resolution parameter is the key parameter. The time complexity of MrTADFinder is subject to $O(n^3)$.

Chen et al. [65] regarded Hi-C matrix as an undirected weighted graph and proposed the Spectral method based on the spectral algorithm. Spectral performs a power transform and a Toeplitz normalization on Hi-C matrix for pre-processing. Spectral calculates the Fiedler vector of the Laplacian matrix to form an initial set of TADs, and then repeatedly computes the Fiedler number and vector from the previously found TADs until the Fiedler number is larger than the threshold, or the TAD size reaches the lower bound. Spectral uses an $n \times n$ Hi-C matrix as input data and outputs non-overlapping TADs.

Cresswell et al. [81] observed the graph-like structure of the Hi-C contact matrix and developed a method based on spectral graph theory, called SpectralTAD. SpectralTAD calls hierarchical TADs via the modified spectral clustering approach with a sliding window to speed up the algorithm and increase the stability. SpectralTAD uses either sparse 3-column, $n \times n$ or $n \times (n+3)$ Hi-C matrix as input data and has one key parameter to specify the hierarchy levels of TADs. The time complexity of SpectralTAD is reduced from $O(n^3)$ to *O*(*n*).

### Data and materials

We partition the datasets into 2 categories: Experimental Micro-C datasets and several synthetic datasets. We used Micro-C datasets to effectively interrogate features below the level of TADs that are largely inaccessible by conventional Hi-C analysis. We retrieve Micro-C datasets over human embryonic stem cells (ESCs) and human fibroblasts from [91] which map chromosome folding at single nucleosome resolution. In these cell lines, we have generated a single sample with the combined sequenced reads by pooling all the replicates. We have also used Micro-C dataset on mouse embryonic stem cells (ESCs) [21]. There are thirty-eight biological replicates from mouse embryonic stem cells (ESCs), which were then pooled after confirming high reproducibility ($>0.95$) across samples. During this text, we may generally use the term Hi-C to refer both Hi-C and its very high nucleosome resolution varint Micro-C experiments.

We mainly use HiCNorm [92] to normalize Micro-C contact matrices, which is an explicit individual-sample approach for Micro-C normalization [92]. HiCNorm introduces a Poisson regression model to correct contact matrix. In HiCNorm, the systematic biases, including fragment length, GC content and sequence mappability, are estimated as a Poisson offset, and the residuals of the regression are regarded as the normalized matrix. We analyze the pooled samples by normalizing interaction matrices 5kb, 10kb resolutions. We have generated subsamples having various sequencing depths according to different ratios (0.01, 0.1, 0.5, 0.9) by randomly downsampling the pooled sample. We have also analyzed the impact of various sequencing depths by generating the normalized interaction matrices at resolutions 5kb and 10kb over the pooled sample. Additionally, we have generated normalized interaction matrices at 1kb resolution over replicate mouse ESCs by KR Normalization [93]. We use these normalized matrices to analyze the outcome in regard to the reproducibility of TADs and biological feature enrichment.

The synthetic datasets are made up of two parts. First of all, in silico realistic Hi-C dataset by Haddad et al. [66]. This dataset has been generated via simulating block copolymers’ interaction frequency with various epigenomic partitions, including 100 interaction maps in total. Secondly, dataset of various noise levels ($0.05-0.2$ with step size = 0.05) and various structures such as nested and non-nested TADs have been generated with a 1kb resolution by the procedure of Forcato et al. [86] over mouse embryonic stem cells.

We obtain structural proteins (SMC3, CTCF, and RAD21), ChIP-seq peaks for Polymerase II, and ChIP-seq peaks for histone marks (H3K4me3, H3K36me3 and H3K9me3) for human embryonic stem cells and human fibroblasts in the hg19 genome from UCSC Encode [94] and NIH Roadmap Epigenomics [95]. We align the housekeeping genes in this research to hg19 genome [96]. Lastly, we obtain SINEs and TSSs files again from UCSC Genome Browser. Similarly, we obtain the same datasets for mouse embryonic stem cells again from from UCSC Encode and NIH Roadmap Epigenomics.

### TAD performance evaluation metrics

We evaluate the TAD-calling methods performance by using 8 metrics as discussed in [88]. Among them, we use Jaccard Index (JI), *p* value ($\le 0.05$) ratio, fold change, boundary tagged ratio, average peak, and TAD$adjR^2$ to evaluate the performance of real datasets. We use the remaining false positive rate (FPR) and true positive rate (TPR) metrics to evaluate the performance on synthetic datasets since both of these metrics require the known ground truth.

We use TAD$adjR^2$ to validate the inferred TADs since contact decay’s regularity is combined with the growing distance in its definition. TAD$adjR^2$ takes high values if TAD partition is good. TAD$adjR^2$ also measures the variability proportion of Hi-C signal as captured via TADs. TAD$adjR^2$ indicator describes how good inferred TADs explain the interaction dataset as well as how good TAD partition in the genome is structured. Additionally, TAD$adjR^2$ indicator is an important measure as large proportion of variability in the interaction frequencies in regard to the distance.

Apart from TAD$adjR^2$, TAD boundaries are also validated by regulatory functional elements throughout the genome which is an important frequently-applied approach in the literature. We validate the distribution of regulatory functional elements with respect to TADs mainly by *p* value ($\le 0.05$), fold change, boundary tagged ratio, the average peak since these elements are depleted or enriched close to TAD boundaries. As a result, the degree of depletion or enrichment of elements can thus be evaluated at different angles by these metrics. Among these metrics, the boundary tagged ratio and average peak show the density and depletion or enrichment frequency better than the other two metrics. The *p* value ($\le 0.05$) ratio and fold change focus on the relative strength across the background. Moreover, we can also calculate Jaccard Index between functional elements and TAD boundaries which can address the average peak metric’s unbalance issue where average peak metric is distorted by the values around known TAD boundaries. Jaccard Index measures the similarity of 2 TAD boundaries sets. We use JI to evaluate the TAD-calling methods concordance as well as TAD boundaries’ reproducibility or similarity. Additionally, we evaluate the results on synthetic data by true positive and false positive rates. The better performance is indicated by a lower false positive rate and higher true positive rate. We provide a more detailed definition of these indicators below:

#### TAD$adjR^2$

Due to the fact that TADs with higher contact frequencies are more likely to be extracted, TAD$adjR^2$ [79] is a measurement of the proportion of Hi-C signal variation which can be explained by TADs at a genomic distance. TAD$adjR^2$ is a modified version of $R^2$ (coefficient of determination) in regression analysis. For a given genomic distance, let $Y_{i}$ denote the contact frequencies of the *i*-th bin, *q* denote the number of bins at the same genomic distance as the *i*-th bin, and *p* denote the number of identified TADs whose sizes are not less than the genomic distance. If the *i*-th bin is in a TAD, ${\hat{Y}}_{i}$ denotes the average contact frequency at the genomic distance within the TAD. If the *i*-th bin belongs to a gap between TADs, ${\hat{Y}}_{i}$ denotes the average contact frequency at the genomic distance in the gap region. ${\overline{Y}}$ is the global average contact frequencies at the genomic distance. Then, given a genomic distance, TAD$adjR^2$ is calculated as

1 $$\begin{aligned} TADadjR^2 = 1 - \frac{(q-1) \sum _{i=1}^{q}(Y_{i} - {\hat{Y}}_{i})^{2}}{(q-p-1) \sum _{i=1}^{q}(Y_{i} - {\overline{Y}})^{2}} \end{aligned}$$

#### Average peak

The average peak [88] is a measurement to describe the density of the occurrence frequency of regulatory elements near the TAD boundaries. Let *n* denote the number of unique TAD boundaries detected in a chromosome and $D_i$ denote the average frequency of occurrence of regulatory elements per 1kb within a 2kb range centered on the *i*-th unique TAD boundary. The average peak is calculated as

2 $$\begin{aligned} {\text {Average peak}} = \frac{1}{n} \sum _{i=1}^{n} D_{i} \end{aligned}$$

#### Boundary tagged ratio

The boundary tagged ratio [55] is a measurement to describe how frequent TAD boundaries enriched for regulatory elements are. Let *S* denote the set of TAD boundaries on which a regulatory element occurs within a centered 2kb range and *n* denote the number of unique TAD boundaries detected in a chromosome. The boundary tagged ratio is calculated as

3 $$\begin{aligned} {\text {Boundary tagged ratio}} = \frac{1}{n}|S| \end{aligned}$$

where |.| means the number of items in a set.

#### Fold change

The fold change [55] is a measurement to describe how much the occurrence density of regulatory elements changes between the region far away from the TAD boundaries and the region near the TAD boundaries. Let $A_i$ denote the average occurrence frequency of regulatory elements per 1kb within a 2kb range centered on the *i*-th unique TAD boundary; let $B_i$ denote the average frequency of occurrence of regulatory elements per 1kb in bilateral regions on both sides 200kb to 500kb from the *i*-th unique TAD boundary, and let *n* denote the number of unique TAD boundaries detected in a chromosome. The fold change is calculated as

4 $$\begin{aligned} {\text {Fold change}} = \frac{1}{n}\sum _{i=1}^{n} \log _{2}(\frac{A_{i}}{B_{i}}) \end{aligned}$$

#### *p* value ($\le 0.05$) ratio

The *p* value refers to the probability of finding the observed, or more extreme, results when the null hypothesis of a study question is true. Here, the *p* value is used to evaluate whether the average peak is significant compared to the background. $A_i$, $B_i$ and *n* are the same as above. Let $C_i$ denote the variance of the occurrence frequency of regulatory elements per 1kb in the range of 200kb to 500kb on both sides from the *i*-th unique TAD boundary. Let *T* denote the set of TAD boundaries whose $A_i$ is significant based on the Gaussian distribution with $B_i$ as the mean and $C_i$ as the variance. The *p* value ($\le 0.05$) ratio is calculated as

5 $$\begin{aligned} p{-}value ( \le 0.05) ratio = \frac{1}{n}|T| \end{aligned}$$

#### Jaccard Index

The Jaccard Index (JI) is also known as the intersection over union and is a measurement of similarity between two finite sets. JI is defined as the size of the intersection set divided by the size of the union set between two finite sets. Let *A* and *B* be two finite sets. Then, JI is calculated as

6 $$\begin{aligned} JI(A, B) = \frac{|A \cap B|}{|A \cup B|} \end{aligned}$$

More specifically, the JI for the similarity of TAD boundaries [86] is based on the bin in which the TAD boundary is located, without shifting to the other bins. Furthermore, JI between TAD boundaries within the $\pm 10$ kb window and regulatory elements is calculated to reflect the degree of intersection of TAD boundaries and regulatory elements. Since the size of TAD boundaries and the size of regulatory elements are not consistent, the denominator in JI is calculated as the sum of numbers of TAD boundaries and regulatory elements, while the numerator in JI is calculated as the number of TAD boundaries and that of regulatory elements, respectively.

#### TPR

The true positive rate is a frequently used metric in machine learning. In a binary classification problem, true positive (TP) samples are the number of positive samples classified as positive samples, false positive (FP) samples are the number of negative samples classified as positive samples, false negative (FN) samples are the number of positive samples classified as negative samples, and true negative (TN) samples are the number of negative samples classified as negative samples. The true positive rate is defined as the quantity of true positive samples divided by the quantity of all positive samples.

7 $$\begin{aligned} TPR = \frac{TP}{TP+FN} \end{aligned}$$

#### FPR

The false positive rate is defined as the proportion of false positive samples to predicted positive samples.

8 $$\begin{aligned} FPR = \frac{FP}{TP+FP} \end{aligned}$$

#### Ranking

For a regulatory element, such as CTCF, the continuous region between the minimum and maximum values of an indicator among all the TAD callers is equally divided into four regions in an ascending order, corresponding to ranking levels (1-4) from the lowest to the highest. The main goal of ranking is to replace the value of an indicator obtained by a method with the corresponding ranking level number.

## Results

### TAD inference across callers, resolutions, and sequencing depths

**Fig. 1:**
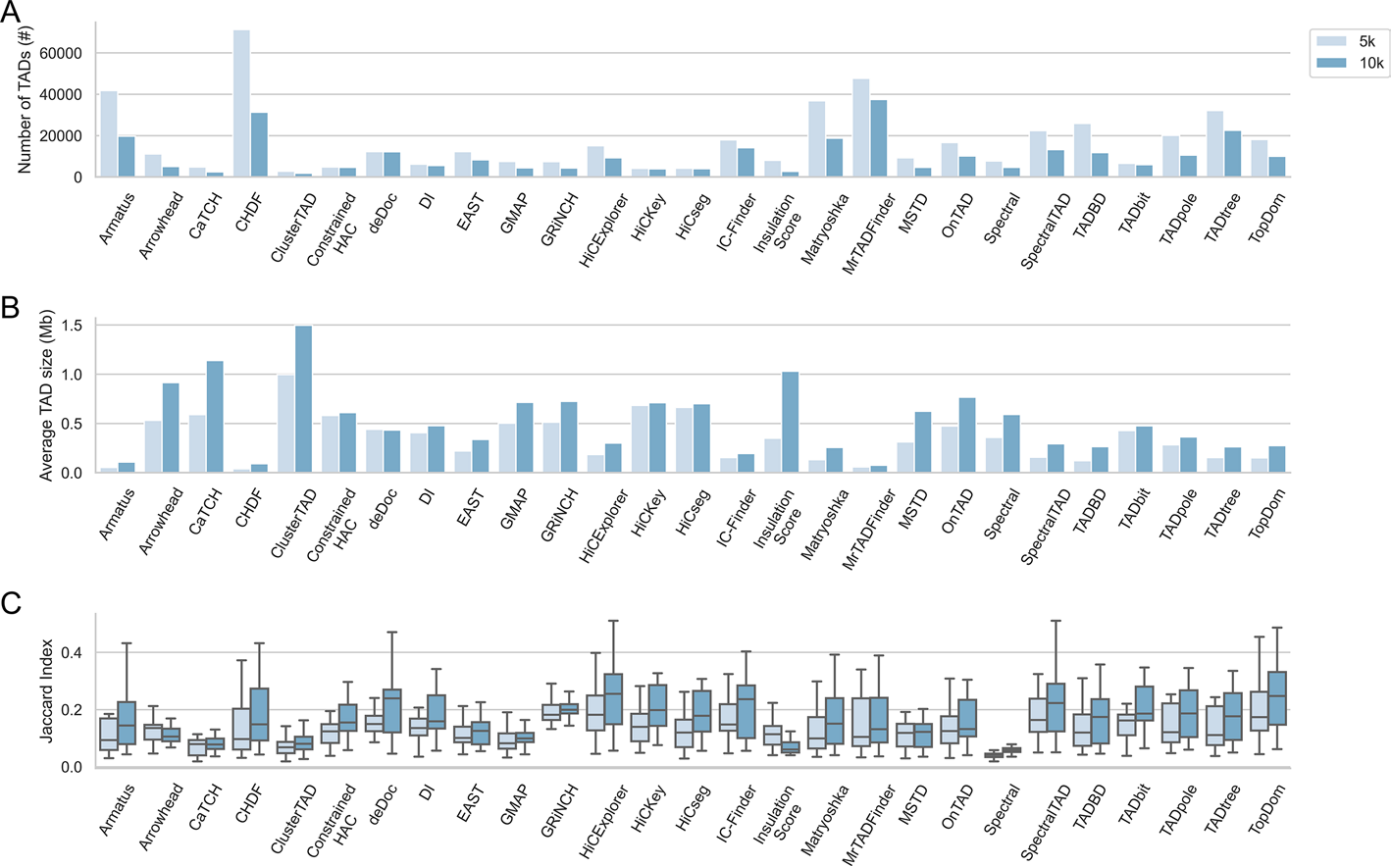
TAD inference performance via TAD callers across a number of interaction matrix resolutions (5kb, 10kb) using HiCNorm normalized Micro-C interaction dataset over human embryonic stem cell line. **A** Across multiple resolutions, the number of TADs inferred via callers in all chromosomes. **B** The mean TAD size inferred by considered TAD callers across different resolutions over all chromosomes. **C** The comparison of topologically associating domain boundaries inferred by a single caller with the remaining callers for a number of resolutions over all chromosomes in terms of Jaccard Index similarity metric

TAD caller, resolution, sequencing depth are key factors that influence the distribution of TADs, on which we assessed the TAD inference performance. We have evaluated the impact of those factors in TAD inference over human ESC cell line. When compared across a number of resolutions (5kb, 10kb), TAD counts decrease but TAD sizes increase for many TAD callers by decreasing resolution as in Fig. 1A, B. Besides TADs’ size and number distribution, TAD inference results’ similarity is also a widely-used assessment factor on TAD inference. For many callers, TAD boundaries’ similarities between a single method and the remaining callers increase by decreasing resolution as in Fig. 1C. As a result, those callers infer more similar topological domains at lower resolutions. Some callers can generate gaps between the inferred topological domains, where the mean gap size increases and the gap count decreases by decreasing resolution.

**Fig. 2:**
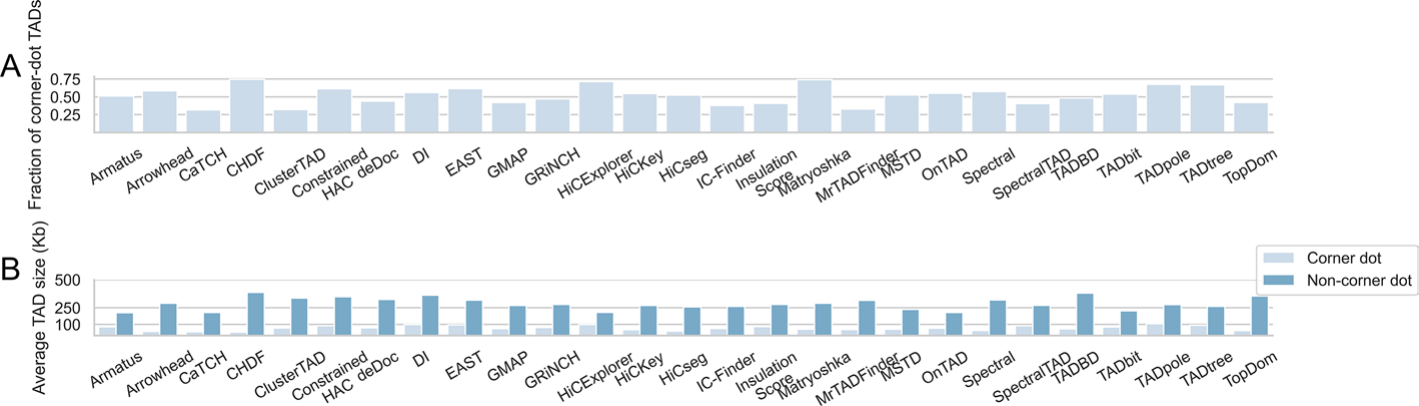
Identification of TADs with and without corner dot (extrusion loop) via evaluated TAD callers over normalized mouse ESCs Micro-C dataset at a lower 1kb resolution. **A** The fraction of TADs with a corner-dot by each caller in all chromosomes. **B** The mean TAD size with and without corner dot, identified by each caller in all chromosomes

Recently, [48] clearly distinguished between large TADs (compartment domains) that look like TADs but lack a corner dot (extrusion loop), and relatively smaller TADs that follow extrusion process properties having a corner dot. Bringing in this perspective, we found feature-based methods such as Armatus, HiCseg, TADbit to identify a more balanced mixture of TADs with and without a corner dot as seen in Fig. 2A over mouse ESCs Micro-C interactions at a lower 1kb resolution, where y axis represents the fraction of TADs with a corner dot. However, certain methods such as CaTCH and ClusterTAD tend to identify larger non-corner dot TADs more frequently, whereas a number of methods such as Matryoskha and CHDF frequently extract smaller TADs with a corner dot. Additionally, average size of non-corner dot domains are in general larger than average size of corner-dot domains across all finders as seen in Fig. 2B.

**Fig. 3:**
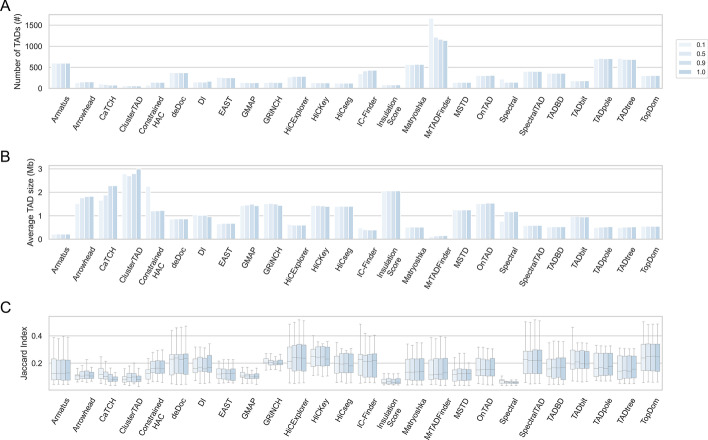
TAD inference performance via TAD callers across a number of sequencing depths at 5kb resolution using HiCNorm normalized Micro-C interaction dataset over human embryonic stem cell line. **A** Across multiple ratio of downsampled sequenced reads, the number of TADs inferred by each caller in all chromosomes. **B** The mean TAD size inferred by considered TAD callers across different ratio of downsampled sequenced reads over all chromosomes. **C** The comparison of TAD boundaries inferred by one TAD caller with the others across a number of sequencing depths over all chromosomes in terms of Jaccard Index similarity metric. We do not report CHDF results as CHDF has inferred each bin in the interaction matrix as an independent TAD after downsampling the dataset

The sequencing depth is an important factor in TAD inference. To explore the impact of sequencing depth, we generate samples where reads are downsampled from pooled samples randomly by a number of ratios (0.01, 0.1, 0.5, 0.9), and investigate the performance of TADs inferred over these generated samples. In contrast to Spectral and MrTADFinder, increasing the sequencing depth increases the quantity of topological domains inferred via IC-Finder and Arrowhead as in Fig. 3. As we increase the sequencing depth, the mean sizes of TADs from Spectral and Arrowhead increase. In terms of similarities of TAD boundaries across different TAD callers, TADs inferred by MrTADFinder and Arrowhead decrease by decreasing the sequencing depth, opposite of Spectral and CaTCH. Among the compared methods, Spectral, CaTCH, MrTADFinder, Arrowhead seem to be affected greater by the sequencing depth, which influences these callers choices about the TAD size and count. The boundaries of TADs inferred over $100\%$ of all reads and TADs inferred over various ratios of downsampled reads become gradually more similar for most callers by increasing sequencing depths as in Fig. 3. According to this figure, for a single TAD-calling method, TAD inference on an interaction dataset with a low sequencing depth will be more biased.

**Fig. 4:**
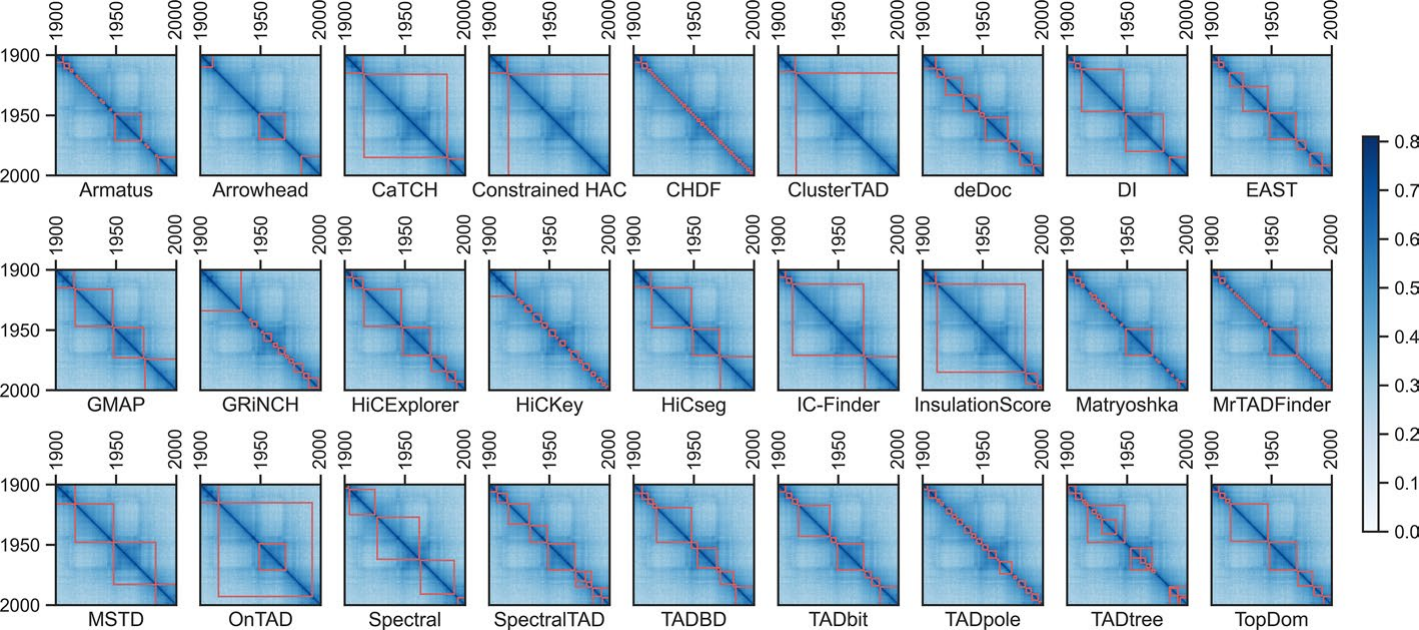
Annotated TADs and heatmaps inferred by TAD callers over the first chromosome region 1900–2000 at 5kb resolution over HiCNorm normalized human embryonic stem cell’s first experiment sample

We differentiate between different TAD-calling methods in terms of the gap size between TADs, the number of gaps between TADs, TAD size, the number of TADs, and similarities of TADs. Practically, TAD-callers have differences in terms of gaps and TADs they generate, which is affected by their different preferences. TAD boundaries identified by TAD caller pairs are not highly similar, which shows the callers different perspective in TAD inference. For instance, heatmaps in Fig. 4 show a part of interaction matrix and TADs (red blocks) over the matrix inferred via each method. Even though a number of TAD-calling methods agree on a portion of TADs, no consensus exists between various TAD-calling methods over the same data.

### Biological assessment of TADs and TAD boundaries

Evaluating TAD callers accuracy is difficult since reaching a consensus agreement for them even on the same data is difficult, and any dataset is not available for labeling and benchmarking. Nevertheless, up-to-date research on TADs have identified the importance of a number of regulatory functional elements and their association with topological domain boundaries [1, 49–52, 54, 55, 97, 98]. Several cohesin complexes such as SMC3 [55] and RAD21, and chromatin insulator CTCF [49–52, 54, 55] are known to contribute to TAD formation where these elements binding sites are also enriched near domain boundaries. Additionally, TAD boundaries are also enriched in terms of histone modifications such as H3K36me3 and H3K4me3, and promoter-related factors such as RNA polymerase II [1, 97, 98]. In contrast, TAD boundaries are depleted in terms of histone modification H3K9me3 [1, 97]. The housekeeping genes (HK genes) [57] and TSSs (Transcription start sites) [1] exist frequently near TAD boundaries. Finally, TAD boundaries in the human genome are enriched especially in terms of Alu SINE elements [1]. When considered all together, these relationships between the regulatory elements and TADs show that TADs are widely-known structures correlated with complex gene regulation in the cell. By analyzing these relationships, extracting extra biological information on the inferred TAD boundaries will be useful in evaluating the TAD-calling methods performance.

**Fig. 5:**
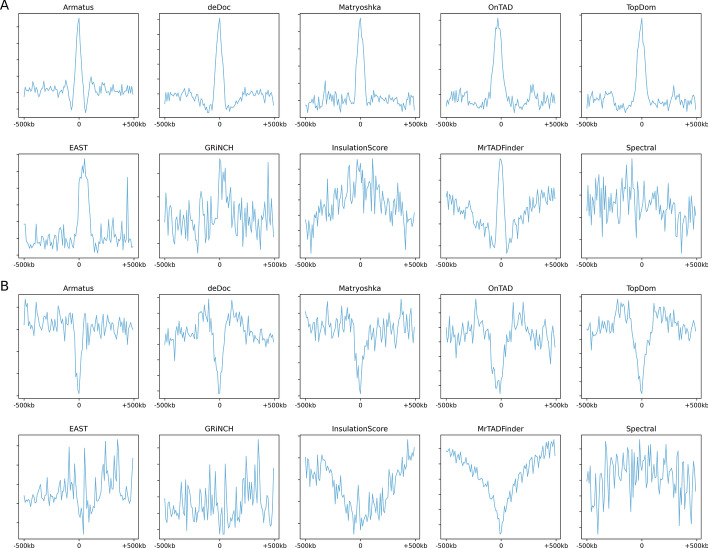
Regulatory elements enrichment and depletion per 1kb near topologically associating domain boundaries with a 1000kb window, identified by different TAD callers at 5kb resolution over HiCNorm normalized human embryonic stem cell’s first experiment sample. **A** CTCF for enrichment and **B** H3K9me3 for depletion. The top rows show the topmost performing 5 callers, whereas the bottom rows show the worst perfoming 5 callers

We calculate the depletion and enrichment of these regulatory functional elements near TAD boundaries by means of 1kb distance from 500kb downstream to 500 kb upstream TAD boundaries regions for all callers. Even though the profiles of depletion and enrichment curves are similar as in Fig. 5, we have calculated *p* value ($\le 0.05$), fold change, boundary tagged ratio, and average peak to investigate the density of these elements’ peaks around topological domain boundaries. The boundary tagged ratio and average peak define the degree of depletion and enrichment of these elements near TAD boundaries. Similarly, we calculate *p* value and fold change to analyze the significance of depletion or enrichment of these regulatory elements. These metrics are already oriented near TAD boundaries. So, we also calculate the JI between regulatory elements and TAD boundaries inside $\pm 10$kb window in order to take into account the bias of depletion or enrichment around TAD boundaries.

The majority of functional regulatory elements are remarkably enriched in terms of average peak metric around TAD boundaries inferred via DI, which is accompanied by feature-based method TADbit and clustering-based method Constrained HAC as in Table 2 over the normalized human embryonic stem cell interaction dataset with 29 replicate samples at the 5kb resolution. The TAD boundaries inferred via HiCExplorer and DI are remarkably enriched for the majority of regulatory elements, which are accompanied by feature-based TADbit according to the boundary tagged ratio as in Table 4. The TAD boundaries inferred via CaTCH are remarkably enriched for the majority of regulatory elements, which is accompanied by Constrained HAC according to the fold change as in Table 3. In terms of *p* value ($\le 0.05$), TAD boundaries inferred via TADtree, TADbit, SpectralTAD, OnTAD, Matryoshka, HiCseg, IC-Finder, DI, CaTCH are significantly enriched around TAD boundaries. The feature-based callers OnTAD and TADtree, and MrTADFinder outperform the remaining callers in terms of JI between the functional elements and TAD boundaries. The ranking column in the tables define the summation of every caller’s ranking for enrichment of the whole set of functional elements except H3K9me3. According to such ranking criteria, the topmost rankings are achieved by OnTAD, HiCseg, HiCExplorer, and DI as in Tables 2, 3 and 4. According to the Tables 2, 3 and 4, in terms of depletion, TAD boundaries inferred via CaTCH is mainly depleted for H3K9me3 according to fold change, boundary tagged ratio, and average peak metrics. In terms of *p* value, TAD boundaries called by almost all callers are significantly depleted in terms of H3K9me3. Lastly, Jaccard Index between TAD boundaries inferred via ClusterTAD and H3K9me3 is the smallest.

**Table 2:** The mean peak near inferred TAD boundaries (2kb) for 10 functional elements over normalized interaction dataset from human embryonic stem cells at 5kb resolution

	H3K9me3	H3K4me3	H3K36me3	CTCF	HK genes	TSS	SMC3	SINE	RAD21	Polymerase II	Rank
Armatus	0.253	0.333	0.337	0.367	0.085	0.646	0.155	4.542	0.144	0.338	16
Arrowhead	0.333	0.401	0.419	0.458	0.108	0.715	0.211	5.366	0.194	0.418	28
CaTCH	0.230	0.371	0.399	**0.238**	0.101	0.650	0.141	3.701	0.126	0.372	14
Constrained HAC	**0.397**	0.451	0.515	0.376	0.111	0.782	**0.271**	5.199	**0.243**	0.503	33
CHDF	0.280	0.339	0.352	0.387	0.084	0.652	0.181	4.482	0.169	0.344	17
ClusterTAD	0.321	0.471	0.505	0.362	0.118	0.832	0.203	5.148	0.187	0.490	31
deDoc	0.345	0.401	0.412	0.416	0.098	0.717	0.229	5.036	0.213	0.406	27
DI	0.375	**0.533**	**0.558**	0.383	**0.139**	**0.916**	0.238	**5.678**	0.216	0.512	**36**
EAST	0.258	0.362	0.388	0.452	0.094	0.694	0.163	4.618	0.153	0.309	17
GMAP	0.287	0.353	0.411	0.413	0.101	0.778	0.174	5.283	0.160	0.420	23
GRiNCH	0.291	0.357	0.415	0.414	0.100	0.775	0.176	5.285	0.158	0.423	23
HiCExplorer	0.393	0.466	0.461	0.417	0.111	0.774	0.258	5.372	0.236	0.463	33
HiCKey	0.332	0.475	0.501	0.344	0.123	0.772	0.197	5.157	0.181	0.490	31
HiCseg	0.330	0.472	0.504	0.347	0.125	0.770	0.201	5.153	0.178	0.489	31
IC-Finder	0.343	0.460	0.458	0.430	0.112	0.790	0.218	5.359	0.201	0.446	28
InsulationScore	0.275	0.365	0.376	0.377	0.101	0.751	0.175	5.429	0.162	0.366	21
Matryoshka	0.274	0.388	0.390	0.365	0.098	0.737	0.173	4.926	0.158	0.370	20
MrTADFinder	0.215	0.248	0.269	0.429	0.067	0.506	0.126	4.354	0.116	0.297	10
MSTD	0.289	0.341	0.358	0.381	0.089	0.656	0.181	4.959	0.159	0.396	20
OnTAD	0.377	0.485	0.479	0.420	0.123	0.826	0.250	5.622	0.231	0.455	35
Spectral	0.191	0.247	0.247	0.419	0.067	0.474	0.118	4.078	0.109	0.246	9
SpectralTAD	0.311	0.384	0.411	0.426	0.095	0.676	0.208	4.840	0.189	0.388	24
TADBD	0.294	0.375	0.387	0.456	0.101	0.724	0.182	5.503	0.172	0.352	21
TADbit	0.361	0.520	0.558	0.410	0.133	0.888	0.224	5.647	0.201	**0.522**	34
TADpole	0.287	0.392	0.390	0.454	0.105	0.725	0.179	5.146	0.165	0.369	21
TADtree	0.284	0.396	0.392	0.453	0.101	0.722	0.177	5.145	0.167	0.367	21
TopDom	0.382	0.467	0.458	0.387	0.110	0.785	0.252	5.316	0.234	0.448	32

Bold numbers show the leading result for every column. The final column is sum of each caller’s ranking in terms of the whole set of functional regulatory elements except H3K9me3

**Table 3:** Fold-change ($\log _{2}$) between bilateral regions (500kb) and TAD boundary segments (1kb) for 10 regulatory functional elements over normalized interaction dataset of human embryonic stem cell line at 5kb resolution

	H3K9me3	H3K4me3	H3K36me3	CTCF	HK genes	TSS	SMC3	SINE	RAD21	Polymerase II	Rank
Armatus	0.318	0.307	0.285	− 0.269	0.324	0.191	0.389	0.038	0.383	0.359	17
Arrowhead	0.609	0.391	0.372	− 0.356	0.473	0.220	0.695	**0.185**	0.646	0.557	27
CaTCH	0.549	**0.706**	**0.731**	**− 0.659**	**0.813**	**0.522**	0.568	0.127	0.471	**0.813**	31
Constrained HAC	**0.873**	0.527	0.663	− 0.409	0.502	0.358	**1.062**	0.132	**0.999**	0.785	33
CHDF	0.481	0.363	0.352	− 0.226	0.309	0.265	0.606	0.036	0.613	0.412	23
ClusterTAD	0.515	0.452	0.495	− 0.474	0.478	0.299	0.598	0.032	0.543	0.577	25
deDoc	0.608	0.314	0.293	− 0.284	0.243	0.183	0.773	0.035	0.745	0.420	20
DI	0.644	0.630	0.616	− 0.438	0.654	0.439	0.714	0.146	0.646	0.721	33
EAST	0.292	0.109	0.233	− 0.077	0.260	0.110	0.372	0.039	0.365	0.062	15
GMAP	0.432	0.291	0.395	− 0.351	0.462	0.292	0.433	0.159	0.381	0.568	24
GRiNCH	0.434	0.294	0.396	− 0.352	0.464	0.295	0.436	0.160	0.382	0.569	24
HiCExplorer	0.696	0.452	0.372	− 0.365	0.332	0.226	0.852	0.082	0.804	0.533	27
HiCKey	0.697	0.454	0.373	− 0.369	0.335	0.228	0.850	0.081	0.802	0.531	27
HiCseg	0.669	0.670	0.699	− 0.405	0.753	0.417	0.689	0.175	0.595	0.810	**34**
IC-Finder	0.532	0.505	0.424	− 0.286	0.408	0.307	0.635	0.096	0.597	0.521	26
InsulationScore	0.274	0.254	0.201	− 0.329	0.394	0.281	0.339	0.178	0.282	0.328	20
Matryoshka	0.365	0.384	0.347	− 0.295	0.394	0.276	0.460	0.075	0.430	0.410	21
MrTADFinder	0.123	− 0.021	0.004	− 0.136	0.047	− 0.042	0.121	0.004	0.098	0.162	9
MSTD	0.491	0.195	0.197	− 0.489	0.243	0.115	0.568	0.111	0.463	0.499	22
OnTAD	0.603	0.428	0.374	− 0.343	0.418	0.273	0.717	0.117	0.689	0.459	26
Spectral	0.041	0.069	− 0.064	0.010	0.108	− 0.045	0.052	− 0.004	0.045	0.046	9
SpectralTAD	0.544	0.425	0.410	− 0.230	0.361	0.221	0.669	0.057	0.634	0.491	24
TADBD	0.213	0.151	0.139	− 0.124	0.191	0.122	0.248	0.117	0.246	0.163	13
TADbit	0.598	0.591	0.642	− 0.377	0.632	0.424	0.657	0.165	0.572	0.747	33
TADpole	0.325	0.327	0.254	− 0.121	0.298	0.199	0.387	0.079	0.380	0.295	18
TADtree	0.323	0.325	0.256	− 0.124	0.297	0.196	0.385	0.077	0.381	0.293	18
TopDom	0.673	0.445	0.353	− 0.382	0.311	0.237	0.830	0.062	0.793	0.505	27

The final column is sum of each caller’s ranking in terms of the whole set of functional regulatory elements except H3K9me3. Bold numbers show the leading result for every column

**Table 4:** The inferred TAD boundaries boundary tagged ratio for 10 functional elements over normalized interaction dataset from human embryonic stem cell line at 5kb resolution

	H3K9me3	H3K4me3	H3K36me3	CTCF	HK genes	TSS	SMC3	SINE	RAD21	Polymerase II	Rank
Armatus	0.695	0.311	0.351	0.630	0.156	0.860	0.570	0.998	0.530	0.501	18
Arrowhead	0.910	0.442	0.512	0.747	0.229	0.911	0.832	0.999	0.796	0.647	32
CaTCH	0.484	0.303	0.341	**0.398**	0.166	0.488	0.448	0.569	0.429	0.417	9
Constrained HAC	0.910	0.469	0.550	0.737	0.248	0.887	0.856	0.987	0.826	0.672	34
CHDF	0.733	0.338	0.383	0.647	0.161	0.849	0.624	0.978	0.590	0.513	20
ClusterTAD	0.779	0.471	0.533	0.689	0.243	0.807	0.712	0.981	0.691	0.679	29
deDoc	0.858	0.435	0.499	0.724	0.218	0.876	0.788	0.996	0.760	0.636	32
DI	0.920	0.530	**0.598**	0.747	**0.289**	0.909	0.858	0.996	0.828	**0.733**	**36**
EAST	0.821	0.404	0.476	0.707	0.200	0.880	0.728	0.992	0.694	0.610	27
GMAP	0.848	0.416	0.482	0.699	0.214	0.883	0.740	0.996	0.704	0.628	30
GRiNCH	0.849	0.419	0.485	0.702	0.216	0.884	0.745	0.998	0.705	0.626	30
HiCExplorer	**0.942**	0.490	0.559	0.770	0.257	0.906	**0.894**	0.997	**0.870**	0.698	**36**
HiCKey	**0.944**	0.492	0.561	0.768	0.259	0.908	**0.894**	0.997	**0.870**	0.699	**36**
HiCseg	0.872	0.505	0.576	0.706	0.278	0.870	0.804	0.988	0.771	0.705	**36**
IC-Finder	0.890	0.476	0.535	0.739	0.239	0.902	0.819	0.999	0.789	0.669	35
InsulationScore	0.828	0.430	0.485	0.681	0.208	0.881	0.719	0.971	0.678	0.640	29
Matryoshka	0.740	0.358	0.403	0.639	0.185	0.873	0.628	0.997	0.590	0.544	22
MrTADFinder	0.702	0.294	0.348	0.704	0.130	0.857	0.567	0.998	0.529	0.508	18
MSTD	0.853	0.380	0.450	0.689	0.194	0.872	0.766	0.991	0.726	0.609	27
OnTAD	0.926	0.507	0.577	0.758	0.263	0.910	0.867	0.998	0.841	0.712	**36**
Spectral	0.665	0.305	0.361	0.649	0.131	0.786	0.548	0.920	0.514	0.477	15
SpectralTAD	0.834	0.399	0.464	0.703	0.200	0.881	0.754	0.998	0.721	0.601	27
TADBD	0.864	0.436	0.499	0.712	0.216	0.901	0.770	**0.999**	0.737	0.630	30
TADbit	0.912	**0.530**	0.596	0.763	0.276	**0.916**	0.844	0.998	0.814	0.731	**36**
TADpole	0.807	0.421	0.471	0.717	0.205	0.885	0.704	0.998	0.673	0.604	28
TADtree	0.805	0.419	0.470	0.716	0.204	0.883	0.703	0.998	0.671	0.600	28
TopDom	0.897	0.471	0.534	0.730	0.242	0.881	0.841	0.996	0.817	0.674	34

Bold numbers show the leading result for every column. The final column is sum of each caller’s ranking in terms of the whole set of functional regulatory elements except H3K9me3

Besides human Micro-C datasets, we have also performed the analysis of enrichment of four typical regulatory elements (CTCF, Housekeeping genes, RAD21, TSSs) near TAD boundaries over Micro-C dataset at a lower 1kb resolution from the mouse ESCs as in Table 5. In this table, we evaluate the performance in terms of 6 metrics: (1) Average peak near inferred topological domain boundaries, (2) Inferred TAD boundaries’ boundary tagged ratio, (3) Fold change ($\log _2$) between bilateral regions and topological domain boundaries, (4) *p* value ($\le 0.05$) of average peak around inferred TAD boundaries in bilateral regions, (5) JI between functional regulatory elements and topological domain boundaries using number of TAD boundaries as numerator, and (6) JI between functional regulatory elements and topological domain boundaries using the number of regulatory elements as numerator, respectively. According to the Table, HiCseg frequently outperforms the other TAD callers in the mouse ESCs in most of the considered metrics. DI method has frequently the topmost rank according to the summation of every caller’s ranking for enrichment of the whole set of functional elements in mouse ESCs.

**Table 5:** Analysis for the enrichment of four regulatory elements (from the left: CTCF, Housekeeping genes, RAD21, TSSs) using the KR-normalized Micro-C dataset ($n = 38$) from mouse ESCs at the 1kb resolution

TAD Callers	Functional regulatory elements				
	CTCF	HK genes	RAD21	TSS	Rank
Armatus	0.334/0.802/0.615/1/0.145/0.772	0.138/0.261/0.54/1/0.19/0.27	0.267/0.756/0.622/1/0.161/0.742	0.517/0.704/0.543/1/0.089/0.836	14/13/10/**16**/13/12
CaTCH	0.261/0.57/0.818/1/0.033/0.684	0.142/0.237/**1.154**/1/0.101/0.488	0.199/0.549/0.681/1/0.039/0.676	0.472/0.508/**1.021**/1/0.02/0.722	10/5/14/16/4/13
Constrained HAC	0.375/0.9/**1.07**/1/0.033/0.622	0.136/0.282/0.937/1/0.089/0.469	0.299/0.875/**1.029**/1/0.039/0.615	0.518/0.693/0.864/1/0.017/0.634	15/14/**16**/**16**/4/10
ClusterTAD	0.351/0.809/0.773/1/0.024/0.473	0.167/0.31/0.954/1/0.083/0.419	0.274/0.775/0.681/1/0.028/0.469	0.587/0.729/0.781/1/0.014/0.511	**16**/14/13/**16**/4/6
DI	0.375/0.917/0.889/1/0.066/0.807	0.175/0.329/1.014/1/0.16/0.481	0.292/0.885/0.798/1/0.077/0.795	**0.603**/0.767/0.911/1/0.037/0.832	**16**/**16**/**16**/**16**/6/**16**
HiCExplorer	0.377/**0.931**/0.917/1/0.075/0.868	0.133/0.271/0.646/1/0.139/0.467	0.311/**0.903**/0.915/1/0.088/0.857	0.525/0.708/0.693/1/0.039/0.881	15/15/13/**16**/7/**16**
HiCseg	0.34/0.889/0.838/1/0.045/0.775	**0.178**/**0.347**/1.137/1/0.136/**0.558**	0.256/0.85/0.726/1/0.053/0.762	0.598/**0.772**/0.98/1/0.026/0.812	15/**16**/15/**16**/6/14
IC-Finder	0.338/0.885/0.747/1/0.097/0.869	0.128/0.255/0.598/1/0.152/0.399	0.275/0.853/0.737/1/0.111/0.85	0.491/0.693/0.626/1/0.052/0.898	14/14/11/**16**/9/15
Matryoshka	0.331/0.857/0.591/1/0.16/0.748	0.14/0.276/0.506/1/**0.203**/0.257	0.266/0.819/0.571/1/0.18/0.72	0.509/0.728/0.509/1/0.095/0.804	13/15/10/**16**/13/11
MrTADFinder	0.183/0.592/0.179/1/**0.231**/0.47	0.081/0.135/0.237/0.286/0.097/0.114	0.143/0.532/0.134/0.857/0.23/0.428	0.325/0.492/0.26/0.857/**0.141**/0.563	4/4/4/12/13/4
OnTAD	0.36/0.925/0.758/1/0.07/0.875	0.146/0.294/0.643/1/0.146/0.496	0.291/0.894/0.732/1/0.082/**0.863**	0.487/0.734/0.539/1/0.037/0.897	14/15/10/**16**/7/**16**
SpectralTAD	0.294/0.804/0.743/1/0.093/**0.879**	0.111/0.213/0.587/1/0.131/0.384	0.237/0.767/0.714/1/0.106/0.856	0.422/0.615/0.608/1/0.049/**0.914**	10/10/11/**16**/8/15
TADbit	0.369/0.902/0.872/1/0.062/0.825	0.17/0.314/0.951/1/0.149/0.495	0.287/0.869/0.788/1/0.073/0.812	0.587/0.758/0.859/1/0.035/0.848	**16**/**16**/15/**16**/6/**16**
TADtree	0.28/0.842/0.412/1/0.22/0.72	0.117/0.242/0.348/1/0.196/0.189	0.227/0.802/0.4/1/**0.243**/0.683	0.416/0.681/0.316/1/0.128/0.795	10/13/7/**16**/**16**/10
TopDom	**0.386**/0.916/0.883/1/0.094/0.873	0.141/0.28/0.605/1/0.163/0.417	**0.316**/0.891/0.878/1/0.11/0.855	0.541/0.73/0.683/1/0.051/0.901	15/15/13/**16**/9/15

For each regulatory element, values from left to right represent (1) Mean peak near inferred TAD boundaries, (2) Inferred TAD boundaries boundary tagged ratio, (3) Fold change ($\log _2$) between bilateral regions and TAD boundary regions, (4) *p* value ($\le 0.05$) of mean peak near inferred TAD boundaries in bilateral regions, (5) JI between functional regulatory elements and TAD boundaries using the number of TAD boundaries as numerator, and (6) JI between functional regulatory elements and TAD boundaries using number of regulatory elements as numerator, respectively. The final column is sum of each caller’s ranking in terms of the whole set of functional regulatory elements. Bold numbers show the leading result for every column

CTCF and Cohesin complex proteins such as RAD21, SMC3 exists more frequently around the boundaries for TADs with corner dot as in Table 6 over the normalized mouse ESCs Micro-C dataset at a lower 1kb resolution. Such abundance is observed slightly more on TADs with corner dot called by feature-based methods. However, distribution of peaks for histone modifications H3K36me3, H3K4me3, and H3K9me3 do not depend on whether TAD have an extrusion loop or not. Such coexistence of CTCF and cohesin related complex SMC3 have been previously discussed in [48, 99–101] where SMC complexes, for instance cohesin and condensin, arrange the chromatin by loop extrusion during the cell phases. The loop extrusion is thought to be a viable chromatin organization mechanism according to [48, 102].

**Table 6:** The mean peak near inferred TAD boundaries (2kb) for six functional elements for both with and without corner dot using the normalized Micro-C dataset over mouse ESCs at a lower 1kb resolution

	H3K9me3	H3K4me3	H3K36me3	RAD21	SMC3	CTCF	H3K9me3	H3K4me3	H3K36me3	RAD21	SMC3	CTCF
	With corner dot (extrusion loop)						Without corner dot					
Armatus	0.451	0.342	0.353	0.531	0.535	0.565	0.221	0.112	0.123	0.301	0.305	0.335
Arrowhead	0.531	0.392	0.409	0.599	0.617	**0.356**	0.301	0.162	0.179	0.369	0.387	0.426
CaTCH	0.428	0.324	0.339	0.569	0.597	0.436	0.198	0.094	0.109	0.339	0.367	**0.206**
Constrained HAC	0.595	**0.441**	0.468	0.649	0.712	0.574	0.365	**0.211**	**0.238**	0.419	0.482	0.344
CHDF	**0.578**	0.367	0.379	0.537	0.551	0.585	0.248	0.137	0.149	0.307	0.321	0.355
ClusterTAD	0.519	0.385	0.401	0.669	0.703	0.561	0.289	0.155	0.171	0.439	0.473	0.331
deDoc	0.543	0.411	0.427	0.599	0.612	0.614	0.313	0.181	0.197	0.369	0.382	0.384
DI	0.573	0.414	0.436	**0.731**	**0.756**	0.581	0.343	0.184	0.206	**0.501**	0.536	0.351
EAST	0.456	0.351	0.361	0.562	0.586	0.652	0.226	0.121	0.131	0.332	0.356	0.422
GMAP	0.485	0.358	0.372	0.551	0.609	0.611	0.255	0.128	0.142	0.321	0.379	0.381
GRiNCH	0.495	0.362	0.375	0.557	0.614	0.615	0.252	0.129	0.144	0.324	0.381	0.382
HiCExplorer	0.571	0.434	**0.476**	0.664	0.659	0.615	0.361	0.204	0.236	0.434	0.429	0.385
HiCKey	0.531	0.377	0.399	0.675	0.705	0.548	0.301	0.147	0.171	0.445	0.475	0.317
HiCseg	0.528	0.376	0.398	0.672	0.702	0.545	0.298	0.146	0.168	0.442	0.472	0.315
IC-Finder	0.539	0.398	0.416	0.658	0.656	0.628	0.309	0.168	0.186	0.428	0.426	0.398
InsulationScore	0.473	0.363	0.374	0.564	0.574	0.575	0.243	0.133	0.144	0.334	0.344	0.345
Matryoshka	0.472	0.356	0.371	0.586	0.588	0.563	0.242	0.126	0.141	0.356	0.358	0.333
MrTADFinder	0.413	0.314	0.324	0.446	0.467	0.627	0.183	0.084	0.095	0.236	0.237	0.397
MSTD	0.487	0.357	0.378	0.539	0.556	0.578	0.257	0.127	0.158	0.309	0.336	0.348
OnTAD	0.575	0.429	0.448	0.683	0.677	0.618	0.345	0.199	0.218	0.453	0.447	0.388
Spectral	0.389	0.307	0.316	0.445	0.447	0.617	0.159	0.077	0.086	0.215	0.217	0.387
SpectralTAD	0.509	0.387	0.406	0.582	0.609	0.624	0.279	0.157	0.176	0.352	0.379	0.394
TADBD	0.492	0.372	0.384	0.573	0.585	0.654	0.262	0.142	0.154	0.344	0.355	0.424
TADbit	0.559	0.398	0.422	0.718	0.755	0.608	0.329	0.168	0.192	0.488	**0.546**	0.378
TADpole	0.483	0.367	0.376	0.596	0.594	0.653	0.351	0.137	0.144	0.365	0.360	0.419
TADtree	0.482	0.365	0.375	0.594	0.592	0.651	**0.352**	0.135	0.145	0.364	0.362	0.421
TopDom	0.485	0.432	0.457	0.665	0.656	0.585	0.255	0.202	0.227	0.435	0.426	0.355

Bold numbers show the leading result for every column

**Fig. 6:**
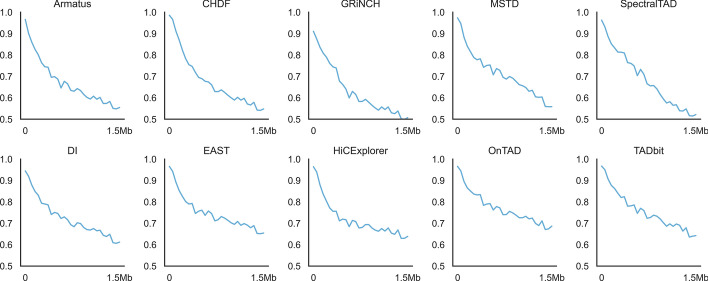
The ratio of Hi-C/Micro-C signal variance over the inferrred TADs as evaluated via TAD$adjR^2$ among a loci pair across $0-1$Mb distance. Multiple TAD callers are evaluated on Micro-C interaction dataset normalized by HiCNorm at 5kb resolution over embryonic stem cell line’s first sample. The top row shows the topmost performing 5 callers, whereas the bottom row shows the worst perfoming 5 callers

Lastly, we also found TADs to keep their biological effects. In terms of TAD definition, TADs having lower interaction frequencies can be rarely identified while TADs having larger interaction frequencies can be more frequently inferred. Additionally, the interaction frequency between each loci pair decay by the increasing genomic distance. In this case, we measure the rate of variability of interaction frequencies described via the inferred TADs throughout genomic distances by TAD$adjR^2$. Figure 6 demonstrates TAD$adjR^2$ profile throughout $0-1.5$M distance over human embryonic cell line’s first replicate at 5kb resolution, where the top row shows the topmost performing 5 callers, whereas the bottom row shows the worst perfoming 5 callers. We have estimated the mean TAD$adjR^2$ across $0-1.5$M distance to analyze the inferred TADs’ quality in the experimental datasets. As seen in Table 7, OnTAD performs better than the remaining callers having the largest mean average TAD$adjR^2$ scores, over normalized human embryonic stem cell interacton dataset of replicates at 5kb resolution. We also observe similar TAD$adjR^2$ scores over human fibroblast interaction dataset at 5kb resolution. As a result, results for the majority of callers are in line with the biological results about topological domains. However, there is no single caller that outperforms all the remaining ones throughout all used metrics.

**Table 7:** The ratio of signal variance over the inferrred TADs as evaluated via TAD$adjR^2$ among a loci pair across $0-1$M distance

	With corner dot (extrusion loop)			Without corner dot		
	Average	Max	Min	Average	Max	Min
Armatus	0.641	0.709	0.524	0.653	0.721	0.535
Arrowhead	0.654	0.740	0.531	0.666	0.751	0.533
CaTCH	0.699	0.759	0.571	0.712	0.773	0.585
Constrained HAC	0.698	0.768	0.568	0.711	0.779	0.579
CHDF	0.654	0.726	0.544	0.667	0.739	0.556
ClusterTAD	0.674	0.741	0.555	0.687	0.755	0.567
deDoc	0.695	0.761	0.573	0.707	0.774	0.585
DI	0.690	0.754	0.568	0.701	0.765	0.582
EAST	0.714	**0.801**	0.583	0.725	**0.813**	0.595
GMAP	0.645	0.712	0.527	0.657	0.725	0.540
GRiNCH	0.648	0.715	0.531	0.659	0.728	0.545
HiCExplorer	0.697	0.766	0.573	0.709	0.777	0.586
HiCKey	0.658	0.735	0.549	0.667	0.745	0.559
HiCseg	0.655	0.733	0.544	0.665	0.744	0.556
IC-Finder	0.685	0.752	0.564	0.696	0.761	0.575
InsulationScore	0.665	0.729	0.549	0.678	0.741	0.561
Matryoshka	0.692	0.761	0.565	0.703	0.774	0.578
MrTADFinder	0.631	0.685	0.535	0.642	0.695	0.545
MSTD	0.671	0.736	0.553	0.683	0.748	0.566
OnTAD	**0.732**	0.799	**0.597**	0.745	0.812	**0.609**
Spectral	0.638	0.711	0.519	0.651	0.722	0.531
SpectralTAD	0.645	0.708	0.533	0.656	0.721	0.545
TADBD	0.686	0.753	0.565	0.696	0.763	0.576
TADbit	0.724	0.793	0.594	0.736	0.805	0.605
TADpole	0.670	0.741	0.559	0.685	0.751	0.571
TADtree	0.672	0.738	0.558	0.682	0.748	0.569
TopDom	0.654	0.717	0.541	0.667	0.729	0.553

Multiple TAD callers are evaluated on the interaction dataset normalized by HiCNorm at 5kb resolution over human embryonic stem cell line. Bold numbers show the leading result for every column

### Concordance and reproducibility of TADs

Besides the biological evaluation of topological domains in terms of functional regulatory elements, concordance and reproducibility of topological domains are major metrics as well while evaluating the TAD callers. While analyzing the concordance and reproducibility of TADs, the experimental dataset is composed of technical and biological replicate samples. Even though TAD boundaries are altered during cell differentiation [38] and development [39, 40] to a certain extent, TADs are still highly reproducible and topological domain boundaries tend to be stable between replicates [1, 3].

**Fig. 7:**
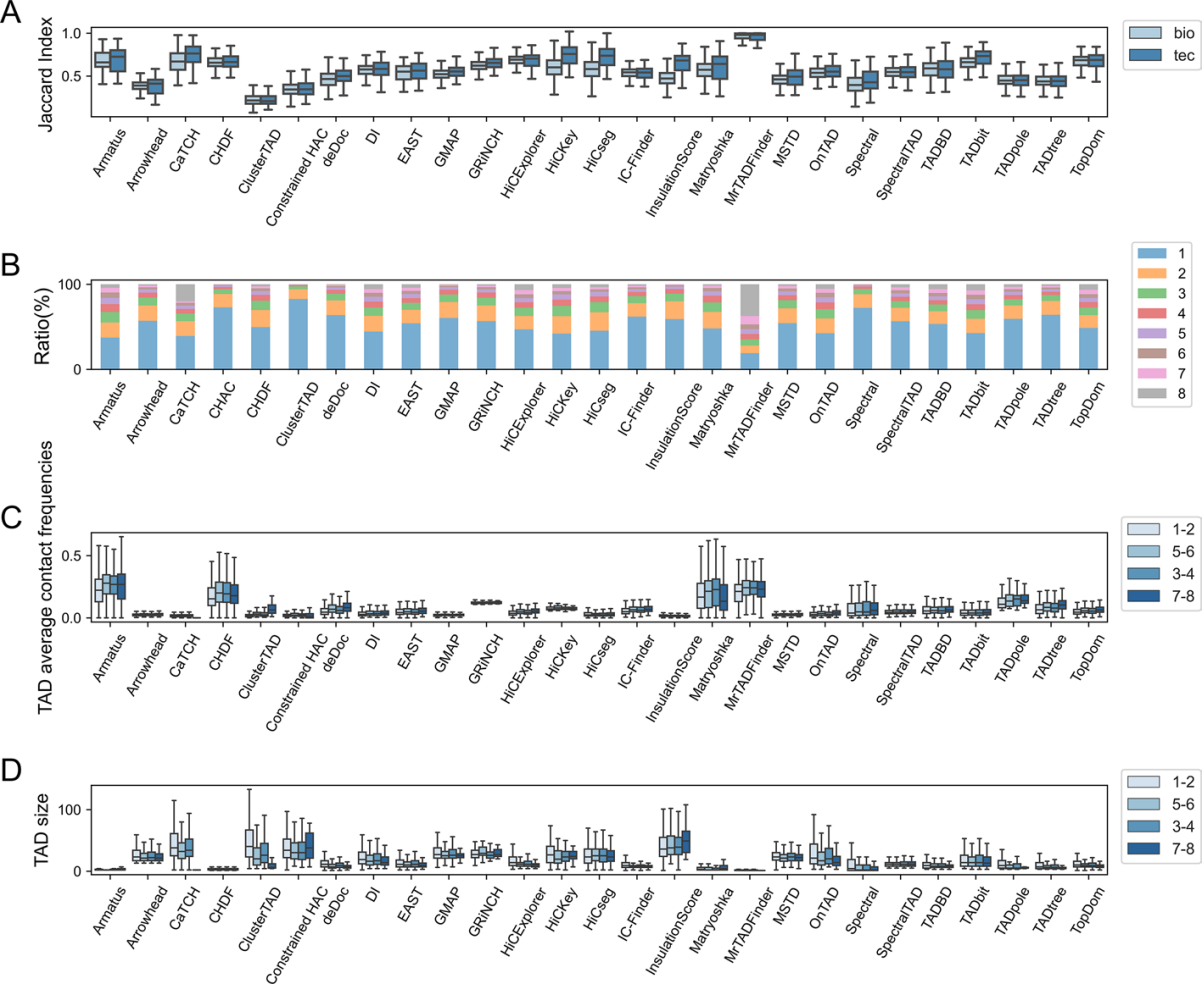
The evaluation of TAD boundaries statistically among the considered TAD callers between replicates over normalized human embryonic stem cell line Micro-C dataset at 5kb resolution, having the same sequencing depths. **A** TAD boundaries reproducibility between technical and biological replicates, as evaluated via Jaccard Index. **B** TAD count distribution relative to various reproducibility levels for individual TAD callers (Levels of reproducibility are between 8 and 1 from top to bottom). **C** The mean TAD interaction frequency across various TAD reproducibility levels between samples. **D** TAD sizes across various TAD reproducibility levels between samples

First of all, we have analyzed the inferred TAD boundaries reproducibility among biological and technical replicates at 5kb resolution in terms of JI, over human embryonic stem cell dataset by replicates with almost identical sequencing depths. As anticipated, TAD boundaries among technical replicates are more reproducible than TAD boundaries among biological replicates as in Fig. 7A over replicates with almost identical sequencing depths. This reproducibility result is also observed across human fibroblast dataset and Micro-C dataset on mouse embryonic stem cells.

The topological domains matching identically by lower an upper boundaries entails the TADs absolute concordance. We have calculated how TAD numbers change across various reproducibility degrees at 5kb resolution over replicates in human embryonic stem cells dataset, where the reproducibility degree defines how many times same TAD occur in the replicates. The majority of topological domains cannot be identified in many replicates simultaneously as seen in Fig. 7B. According to this figure, the majority of topological domains identified by one TAD-calling method on any replicate are less probable to be common across the remaining replicates. Such result is also observed across human fibroblast dataset and Micro-C dataset on mouse embryonic stem cells. Among the callers, feature-based method Armatus and graph-based method MrTADFinder infer topological domains with higher reproducibility than remaining callers. Finding a substantial reproducible set of TAD boundaries by one caller supports the previous findings that topological domains are greatly conserved. Evidently, we have found that smaller and highly-interacting domains tend to be mostly related to robust TADs.

Moreover, to understand the existence of any prospective pattern in TAD inference, we have analyzed the size and mean interaction frequency of topological domains at various reproducibility degrees. The mean interaction frequencies of domains that are highly reproducible are significantly greater than the mean interaction frequencies of domains that have low reproducibility as in Fig. 7C according to Wilcoxon signed-rank test at $p < 0.05$ threshold. As a result, we found that topological domains that have low mean interaction frequencies are less robust than domains having higher mean interaction frequencies. The mean interaction frequencies of topological domains differ less for TAD-calling methods which performance is further balanced as mentioned previously. As opposed to this result, the mean TAD size that have lower reproducibility is greater than the mean TAD size with higher reproducibility for many methods as in Fig. 7D. This result might show that large-scale topological domains are less robust than small-scale domains. These discoveries are also observed across human fibroblast dataset and Micro-C dataset on mouse embryonic stem cells. According to this analysis, smaller and highly-interacting TADs are highly reproducible, which is in line with TAD definition.

### Assessment in simulations

Although labeled topological domains are not provided by the experimental datasets, labeled TADs are available in synthetic datasets and they can be utilized to evaluate TAD-calling methods performance. By using synthetic datasets, we can also evaluate TAD-calling methods performance across various data categories such as without or with nested TADs and noise levels.

**Fig. 8:**
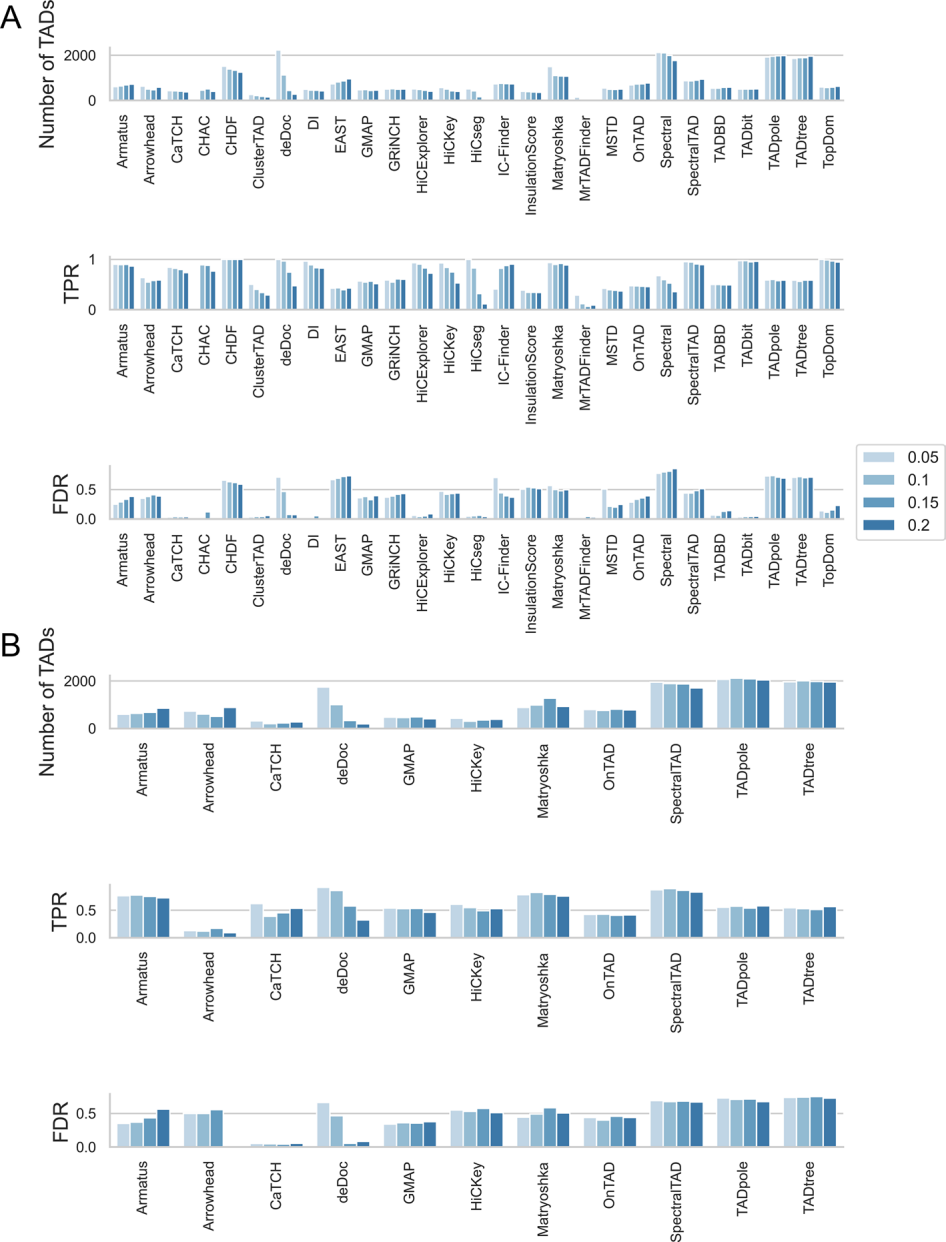
Different TAD callers performance using simulated dataset with various noise levels. **A** The total number (top), TPR (middle), and FDR (bottom) of TADs from non-nested TADs simulated interaction dataset (dashed lines indicate labelled TADs of the simulated data). **B** The total number (top), TPR (middle), and FDR (bottom) of TADs from the nested TADs simulated data

First of all, we have generated the synthetic datasets by mimicking mouse embryonic stem cells’ Micro-C interaction matrix for chromosome 5 at 1kb resolution via Forcato et al.’s method [86]. We have also repeated the same procedure on the remaining chromosomes and we found the results to be highly similar. Among the callers, deDoc and HiCseg are highly sensitive to noise as the TAD counts for both methods have consistently dropped at high noise degrees as seen in Fig. 8. Then, we have evaluated the TAD boundaries in terms of FDR and TPR. CHDF outperforms the remaining callers in accurate inference of TAD boundaries over both nested and non-nested synthetic data in terms of TPR. As the noise levels increase, TPR scores for plenty of callers decrease, such as HiCseg, HiCExplorer, deDoc. On the other hand, TPR scores for a number of callers slightly decrease showing the capability of these callers in supressing a certain noise level, such as TADbit, IC-Finder, and OnTAD. According to FDR, EAST, TADtree, and Spectral infers the most of incorrect TAD boundaries. As noise levels increase, FDR scores for numerous callers, such as EAST and Armatus, increase. At higher noise levels, IC-Finder performs the best in calling hardly any incorrect TAD boundaries. This finding at a higher levels of noise can also be explained by smaller TADs being more connected and decreasing the number of inaccurate TAD boundaries. Finally, feature-based caller TADbit’s performance is more balanced with lower FDR and higher TPR for TAD boundaries on the synthetic datasets. Additionally, the topological domains inferred over synthetic datasets with non-nested domains are more similar to labeled domains than topological domains inferred over synthetic datasets with nested domains.

Secondly, we have simulated the block copolymers interaction frequency with various epigenomic partitions, and generated in-silico datasets. We have evaluated the performance in terms of FDR and TPR of TAD boundaries as in Table 8. According to the table, on average, the domain boundaries inferred via CHDF is the topmost in terms of TPR whereas the domain boundaries inferred via TADBD is the lowest in terms of FDR. Practically, the performance of IC-Finder and TopDom is more balanced than the remaining callers as they exhibit lower FDR and higher TPR over in-silico dataset.

**Table 8:** TAD boundaries’ True Positive Rate and False Positive Rate called from the in silico dataset

	True Positive Rate (TPR)			False Positive Rate (FPR)		
	Average	Max	Min	Average	Max	Min
Armatus	0.708	0.917	0.462	0.192	0.438	0.041
Arrowhead	0.218	0.35	0.141	0.586	0.703	0.444
CaTCH	0.194	0.471	0.044	0.052	0.375	**0**
CHAC	0.355	0.5	0.233	0.047	0.179	**0**
CHDF	**0.965**	**1**	0.81	0.381	0.517	0.237
ClusterTAD	0.354	0.453	0.244	0.153	0.375	**0**
deDoc	0.497	0.632	0.372	0.28	0.46	0.068
DI	0.113	0.198	0.041	0.864	0.95	0.754
EAST	0.26	0.343	0.014	0.539	0.636	0.417
GMAP	0.255	0.412	0.136	0.425	0.6	0.182
GRiNCH	0.341	0.504	0.217	0.327	0.562	0.113
HiCExplorer	0.877	0.949	0.709	0.002	0.036	**0**
HiCKey	0.316	0.429	0.215	0.018	0.089	0.012
HiCseg	0.275	0.406	0.181	0.006	0.071	**0**
IC-Finder	0.946	**1**	**0.848**	0.077	0.158	0.025
InsulationScore	0.09	0.203	0.024	0.636	0.875	0.333
Matryoshka	0.706	0.875	0.5	0.332	0.606	0.127
MrTADFinder	0.752	0.895	0.557	0.337	0.594	0.107
MSTD	0.264	0.383	0.174	0.053	0.211	**0**
OnTAD	0.429	0.487	0.367	0.014	0.094	**0**
Spectral	0.182	0.425	0.058	0.785	0.887	0.571
SpectralTAD	0.869	0.956	0.684	0.213	0.367	0.089
TADBD	0.482	0.5	0.38	**0**	**0**	**0**
TADbit	0.452	0.571	0.304	0.478	0.653	0.313
TADpole	0.431	0.522	0.345	0.216	0.521	0.128
TADtree	0.961	**1**	0.785	0.001	0.016	**0**
TopDom	0.708	0.917	0.462	0.192	0.438	0.041

Bold numbers show the leading result for every column

TAD callers perform better over synthetic dataset with non-nested topological domains than the dataset with nested domains, which can be explained by the interaction dataset’s complexity. According to these findings, experimental interaction dataset is composed of hierarchical TADs so topological domain inference is a challenging problem. Apart of these findings, the degree of noise also has an impact on TAD-calling methods performance. Different than the experimental dataset, synthetic dataset generation takes into account only certain factors so the results on synthetic datasets might solely reveal certain features of those callers up to a certain degree.

### Implementation assessment

Every compared TAD-calling method is available publicly either as a software or web service. It is important for implementations to be easy to be used. But, the runtime of the callers is also very important since computer resources are costly.

We have evaluated the runtime of various callers by calling them over a single chromosome. The runtime has changed between the callers. We have recorded the runtime of callers under predetermined parameters at 5kb resolution over human embryonic cell line’s chromosome 6 as seen in Table 9. TADtree takes the longest to infer TADs, whereas feature-based OnTAD consumes the least amount of time in TAD inference. OnTAD takes less amount of time since OnTAD’s inference complexity does not depend on interaction matrix size *n*, and terms appearing in the complexity are in general less than *n*. On the other hand, the runtime complexity of TADtree is the highest among all callers. The input data in experiments is a sparse large interaction matrix with $n=32442$ and $75.25\%$ zero entries. In general, the reported runtimes are in line with these callers theoretical complexity. One exception is deDoc which runs slower than its expected complexity $O(n\log ^{2}n)$. Such mismatch can be explained by: 1- deDoc’s greedy strategy might be impacting its inference negatively, 2- deDoc runs its internal function twice which is subject $n\log ^{2}n$. The different callers have been implemented by different programming languages which change in their runtime characteristics. In the future, we still expect a caller to infer TADs via using less resources in a reasonable amount of time. Evidently, a caller having higher complexity can only process low resolution datasets which prevents them from inferring TADs over high-dimensional matrices.

**Table 9:** Running time of different TAD callers at 5kb resolution for chromosome 6 on human embryonic stem cell line, tested over a 2.9 GHz 8-Core Intel i9 cpu having 128 GB memory

Methods	Key Parameters	Running time (s)
Armatus	g = 0.5	90.76
Arrowhead	Normalization = KR	92.93
CaTCH	Defaults	44.19
Constrained HAC	h = 342	26.48
CHDF	length = 3422; number = 3422; size = 342	952.38
ClusterTAD	Max_TADsize = 800000	7608.69
deDoc	Defaults	264.85
DI	window = 2500000; min = 2; prob = 0.99	294.77
EAST	Defaults	11.28
GMAP	Defaults	62.46
GRiNCH	Defaults	22.57
HiCExplorer	minDepth = 3*50000; maxDepth = 6*50000; step = 50000	22.94
HiCKey	Defaults	42.13
HiCseg	change_max = 342; distrib = ‘G’;	146.32
IC-Finder	Defaults	64.49
InsulationScore	is = 2500000; ids = 1000000; im = mean; bmoe = 3; nt = 0.1	474.39
Matryoshka	g = 0.5	123.95
MrTADFinder	res = 1.5	625.73
MSTD	MDHD = 10; window = 10	16.48
OnTAD	Defaults	4.78
Spectral	Threshold = 0.8; region size = 1	61.69
SpectralTAD	levels = 2	32.15
TADBD	Defaults	19.19
TADbit	Defaults	20910.81
TADpole	Defaults	211.63
TADtree	S = 50; q = 12; p = 3; M = 10; N = 1025; gamma = 500	32732.71
TopDom	w = 5	28.43

## Discussion

We obtain different results for the compared TAD-calling methods since callers are run on different datasets with different parameters. It is a challenging task to fairly evaluate the existing callers effectiveness. Nonetheless, the compared callers in general come up with similar results by using regularized sample attributes and reasonable parameters. We use the same parameters for the whole set of callers in our comparisons. So, we have not stressed out the diversity of the findings across various parameters.

Numerous callers have recently been proposed to extract TADs with the advent of interaction datasets, which is helping us to better analyze the fundamental genome structure. First of all, we have analyzed the impact of sequencing depth and resolution on the inferred topological domains. The size and number of TADs, and gaps between nearby topological domains can be modified uniformly by the resolution parameter for most callers. Different TAD-calling methods have different sensitivities, so inference results from various sequencing depths are not uniform in the size and number of topological domains. Both the sequencing depth and resolution generally have an impact on TAD boundaries similarity. While a number of TAD-calling methods vary in their inference standards, different TAD partitions from these TAD-calling methods are praiseworthy to the following genome analysis because of TAD variability. Even though a gold standard dataset over TADs for benchmarking purpose is missing, we can still evaluate the callers performance by TADs specificity and quantity, for instance standards for Hi-C signal variation and TAD boundaries. As a result of missing benchmark for TAD inference, verification of the TADs biological features appears to be one meaningful direction to analyze whether domains are inferred incorrectly. Certain TAD-calling methods perform better than the remaining ones when evaluated in terms of 1- validating the association between topological domain boundaries and known regulatory functional elements, 2- validating by TAD$adjR^2$ which measures the ratio of Hi-C signal variability that can be described by topological domains. Since there are numerous evidences showing that topological domains behave as genome’s stable composition, a robust caller might infer significantly reproducible domains in replicates. TAD boundaries on average are less reproducible between biological replicates than between technical replicates. Smaller topological domains with high interaction frequencies are highly reproducible across TADs with various reproducibility between replicates. Moreover we have analyzed the TAD-calling methods performance by comparing the domains inferred via various callers over labeled domains on simulated dataset. Particularly, topological domains inferred from synthetic data not having nested TADs perform superior than that inferred from synthetic data having nested TADs, which shows it is highly probable that the callers do not accurately infer hierarchical TADs. Overall, the callers that are developed to infer hierarchical domains frequently perform worse than the callers that are not developed to call hierarchical domains since inferring hierarchical domains is more difficult than inferring non-hierarchical domains.

We have explored the reason behind the remarkable differences between different TAD-calling methods, particularly according to reproducibility, size, and number of TADs. In this case, making a number of assumptions about the hierarchy, size and number of TADs might bring about different topological domains. Additionally, employing different factors in determining the final topological domains might also bring about different topological domains. Some sample factors are the window size to calculate the mean interaction frequencies between the downstream and upstream bins, and false positive threshold while removing false positive topological domains.

Among 3 caller categories, feature-based callers utilize concise techniques, work on larger-scale, and they have broad quality capturing chromosome interactions. These methods are effective in inferring the patterns and features of TADs from interaction data, and in integrating statistical techniques into inference. On the other hand, clustering methods generally merge adjacent bins by similarity in TAD inference, which is a significant contribution to the TAD callers. Finding a criterion in determining the relevant clusters is one of the difficulties in clustering techniques, which is handled mainly by assumption of experience. Lastly, graph-partitioning callers utilize the graph-based techniques in inferring topological domains by treating interaction matrix as a graph’s adjacency matrix, without integrating many TAD patterns and TAD features in the interaction data. According to our assessment of the inferred topological domains, feature-based callers frequently outperform the remaining callers which suggests the significance of TAD patterns and TAD features in the interaction data. Nevertheless, a number of existing callers are limited in TAD inference such as them fixing the window size limits the topological domains probes and misses the global knowledge. Another issue may be the parameter that decides the maximal TAD number, which will be especially difficult for users that do not have knowledge on experimental datasets apriori.

On top of TAD-calling methods, Hi-C and Micro-C datasets are also concerning in TAD inference. We expect simulated datasets having labels to help in accelerating the TAD inference since experimental dataset with labels do not exist. Even though the simulated datasets try to imitate the experimental datasets, it is still open whether simulated datasets distribution is consistent in regard to the distribution of the experimental datasets. The reliable generation of simulated interaction datasets that mimic the distribution of experimental datasets is a challenging problem. Lately, Generative Adversarial Network (GAN)-based models [103–106] has appeared which can better learn the generation of synthetic datasets across super-resolution, such as the distribution of the generated high-resolution dataset is more consistent with that of low-resolution data. According to these models, GANs can be a great tool in generating reliable simulated datasets which can better mimic the experimental datasets while also being labeled. Since the availability of reliable and consistent synthetic interaction datasets also lead to significant enhancements in TAD inference, callers will not be restricted to unsupervised learning techniques.

In this research, we have compared 27 TAD callers performance across various sequencing depths and resolutions, including their concordance, reproducibility, and quality over synthetic and experimental datasets, and also analyze their performance in terms of callers features. We have also analyzed several potential challenges in TAD inference and also discussed the enhancement direction of the developed techniques. Centered on our systematic comparison of callers, we have come up with a framework to enhance the TAD-calling methods quality by taking implementation efficiency, practicality, reproducibility, and quality into account. In comparison to the current reviews [53, 55, 86, 88], the major motivation behind this study is to develop a systematic, comprehensive, and concise framework to evaluate the TAD-calling methods performance. We also provide these callers implementations in order to compare the performance of callers as quick as possible.

As a future work, we come up with the subsequent views in order to advance 3D genomics area. First of all, TAD inference quality can be enhanced. At the initial step, extracting patterns and decreasing the datasets noise levels are critical steps in improving topological domains quality as the experimental datasets are already noisy. Since many enhanced normalization techniques [107–111] have already been applied to decrease the interaction datasets noise, a number of novel deep learning-based techniques such as ZSSR (“Zero-Shot” Super-Resolution) model [112] can be applied to decrease the datasets noise. Graph-partitioning methods are good candidates for methodological enhancement since they can probe the fundamental structures in genome via global knowledge of interaction data as well as these methods have recently started to receive more frequent attention in TAD identification. Graph-partitioning methods could take the global knowledge on the interaction dataset into account, when compared to the feature-based approaches. Additionally, almost all of the graph-partitioning methods do not need the number of clusters to be provided as apriori.

Furthermore, the relationship between topological domains and gene regulation can be analyzed as well. Numerous functional elements are found to be closely related to topological domain boundaries, which in turn lead to the conformation of stable structure in gene expression and regulation [1, 97, 98]. But, many open questions still exist in analyzing the relationship between TADs and gene regulation. Joint analysis of inferred TADs with additional genome analysis is significant in explaining the associations between different functional regulatory elements and gene regulation mechanisms, including replication activities, translation, and transcription. Additionally, such joint analysis is useful in capturing how expression of genome functions are affected by 3D chromosome structure. For instance, more comprehensive and broad analysis of ChIP-seq might be carried out around topological domain boundaries.

Moreover, topological domains can be used to explain phenotypes. To a certain extent, topological domain boundaries and topological domains are not static since TADs differ across species, cell types, and cell cycle phases. Cancers, gene mutations, and genetic diseases modify the topological domain boundaries which accordingly change the phenotypes. Additional research on differential gene expression across different cell phases or cell types can be carried out to better understand the mechanism by which genes influence phenotypes. We can also carry out control experiments for topological domain inference to find disease related genes. TADs are principal spatial genome structures, they regulate the normal cell development, and their analysis will be important in genomics.

## Conclusion

As a summary, inferring topological domains from interaction datasets is a computational challenge. Almost all TAD callers are grounded on the assumption that the interaction frequencies of the same topological domain are denser than the interaction frequencies between bins that are not in the same topological domain. Even though TAD inference assumption is similar across the callers, numerous callers have been developed to infer topological domains from different perspectives. We propose a common comparison framework to evaluate the inferred TADs quantitatively, we assess different TAD callers performance, and we have extracted their features and preferences. Even though some callers have been outperformed by the remaining ones in our analysis, each proposed caller have brought insights into TAD inference, jointly advanced the TAD inference development, and have disclosed compelling discoveries about topological domains. This work is intended to reveal the existing state of TAD inference and clarify potential challenges. In the meantime, TAD inference is an area of development, constant renewal, and modification. TAD inference has not currently been rigorously studied, and significant amount of research should be carried out over it.

## Data Availability

Data and source code of this research are available on https://github.com/seferlab/TADcomparison.
